# An Evaluation of Traditional Persian Medicine for the Management of SARS-CoV-2

**DOI:** 10.3389/fphar.2020.571434

**Published:** 2020-11-25

**Authors:** Roodabeh Bahramsoltani, Roja Rahimi

**Affiliations:** ^1^ Department of Traditional Pharmacy, School of Persian Medicine, Tehran University of Medical Sciences, Tehran, Iran; ^2^ PhytoPharmacology Interest Group (PPIG), Universal Scientific Education and Research Network (USERN), Tehran, Iran

**Keywords:** herbal medicine, coronavirus, Traditional Persian medicine, antioxidant, phytochemical

## Abstract

A new coronavirus causing severe acute respiratory syndrome (SARS-CoV-2) has emerged and with it, a global investigation of new antiviral treatments and supportive care for organ failure due to this life-threatening viral infection. Traditional Persian Medicine (TPM) is one of the most ancient medical doctrines mostly known with the manuscripts of Avicenna and Rhazes. In this paper, we first introduce a series of medicinal plants that would potentially be beneficial in treating SARS-CoV-2 infection according to TPM textbooks. Then, we review medicinal plants based on the pharmacological studies obtained from electronic databases and discuss their mechanism of action in SARS-CoV-2 infection. There are several medicinal plants in TPM with cardiotonic, kidney tonic, and pulmonary tonic activities, protecting the lung, heart, and kidney, the three main vulnerable organs in SARS-CoV-2 infection. Some medicinal plants can prevent “humor infection”, a situation described in TPM which has similar features to SARS-CoV-2 infection. Pharmacological evaluations are in line with the therapeutic activities of several plants mentioned in TPM, mostly through antiviral, cytoprotective, anti-inflammatory, antioxidant, and anti-apoptotic mechanisms. Amongst the primarily-introduced medicinal plants from TPM, rhubarb, licorice, garlic, saffron, galangal, and clove are the most studied plants and represent candidates for clinical studies. The antiviral compounds isolated from these plants provide novel molecular structures to design new semisynthetic antiviral agents. Future clinical studies in healthy volunteers as well as patients suffering from pulmonary infections are necessary to confirm the safety and efficacy of these plants as complementary and integrative interventions in SARS-CoV-2 infection.

## Introduction

Coronavirus 2019 (SARS-CoV-2) is a new member of the Coronaviridae family which has caused a global outbreak of a disease called COVID-19 (Wu et al., 2020). Despite the current pharmacotherapies, including different antiviral agents used for the management of hospitalized patients like remdesivir, lotinavir, ritonavir, and ribavirin, a growing number of deaths still occur all over the world (World Health Organization (2020) situation report 197) which has made scientists seek better therapeutic agents. Plants have always been an important source of medicinal ingredients, including antiviral agents (Lin et al., 2014; Bahramsoltani et al., 2016). Although there is no approved drug for this disease, it is worth mentioning that oseltamivir (Tamiflu), an antiviral agent currently used in some SARS-CoV-2 infected patients, is based on shikimic acid from *Illicium verum* Hook.f. (star anise) fruit as the precursor (Patra et al., 2020). Additionally, the clinical effectiveness of several medicinal plants such as licorice and garlic has been demonstrated in the treatment of severe acute respiratory syndrome coronavirus (SARS-CoV) and Middle East respiratory syndrome coronavirus (MERS) epidemics (Xiu-hui et al., 2003; Chen and Nakamura, 2004; Cui et al., 2020). A dramatic reduction of mortality from 52% to 1-4% was observed during the SARS epidemic in Beijing due to the addition of Traditional Chinese Medicine to conventional therapies (Chen and Nakamura, 2004). Thus, plant-derived natural agents provide a valuable list of compounds with possible antiviral properties against SARS-CoV-2 which can be the focus of future investigations. Furthermore, the use of complementary and alternative medicine, including different traditional medicines, can be cost-saving and decrease the prescription of conventional drugs, providing further reasons for scientists to undertake studies on natural products (World Health Organization (2013) WHO traditional medicine strategy: 2014-2023).

Traditional Persian Medicine (TPM) is one of the most ancient medical doctrines mostly known through manuscripts by Persian scientists such as *The Canon of Medicine* by Avicenna and *The Great Continens* by *Rhazes* (Hamedi et al., 2013). Additionally, TPM owes several other scientists with valuable manuscripts regarding anatomy and physiology, disease diagnosis, surgery instruments, and single and compound natural medicines (El-Seedi et al., 2019). TPM has several recommendations for the management of organ damage due to various infections. One of the main approaches in the primary and secondary treatment of diseases in TPM is to protect the four body humors, which are blood, phlegm, bile, and melancholy, from infection (Emtiazy et al., 2012; Kopaei et al., 2016). Many types of infections or the so-called “humor infections” with different clinical manifestations have been described in TPM manuscripts and textbooks, some of which have similar features to that of SARS-CoV-2. One of the pathological conditions explained in TPM for the humors is “humor excitation” which is equivalent to the activation of inflammatory pathways (Aghili Khorasani, 1771).

An examination of pathological conditions similar to COVID-19 along with the therapeutic approaches that are described in TPM manuscripts could pave the way for designing a series of natural products for the management of SARS-CoV-2 infection and related complications (Siahpoosh, 2020). Taking concepts from TPM to treat severe infective pulmonary disorders can help us to select medicinal plants that are potentially useful for SARS-CoV-2 (Kenari et al., 2018). The TPM approach includes lifestyle modifications, along with the administration of some medicinal plants to modify the quality and quantity of the four humors from a pathologic situation into the physiologic condition. The main organs affected in COVID-19 are the lung, heart, and kidney (Su et al., 2020; Zheng et al., 2020). Accordingly, another approach, which extracts TPM suggestions for the management of the disease is to focus on the natural agents that are specifically recommended as a tonic for these three organs. A “tonic”, e.g. a cardiotonic medicine, in TPM is defined as a medicine by which an ideal condition is provided for the physiological functions of an organ so that it is less vulnerable to the pathological conditions (Aghili Khorasani, 1771). This study aims to introduce some of the medicinal plants that TPM claims are effective in the management of symptoms similar to COVID-19 and present current evidence on their efficacy.

## Methods

This study used several texts, including the *Makhzan-al-Adviah*, written by MH Aghili Khorasani in 1772 A.D. (Aghili Khorasani, 1771 AD), which is the most recent and complete encyclopedia of medicinal materials in TPM, as well as *The Great Continens* (Rhazes, 960) and *The Canon of Medicine* (Avicenna, 1025). We searched these books/manuscripts for references to medicinal plants that are described as having protective properties against humor infection and excitation, as well as tonifying and protecting effects on the lungs, heart, and kidney.

Our interpretation and translation of old Persian names into scientific names was based on a book by Ghahreman and Okhovvat (2009), which provides accounts of the most relevant scientific names according to morphological descriptions. The list of plants retrieved during this process was then searched in electronic databases including PubMed, Scopus, Cochrane Library, and Web of Science. Data were collected from inception until March 2020. Only published articles were included in this review and unpublished works were not considered. Language restriction was performed, and English language articles were included. The search terms were the scientific names and common names of each plant combined with “antiviral”, “influenza”, “lung”, “pulmonary”, “alveolar infiltration”, “cardiac”, “heart”, “cardiomyopathy”, “renal”, “kidney”, or “immunomodulatory”. The inclusion criteria were any *in vitro*, *in vivo*, or clinical evidence on the antiviral activity of the selected plants against human pathogenic RNA viruses. Additionally, any study about the beneficial effects of these plants or their isolated phytochemicals on the heart, lungs, and kidneys was included. Studies on animal/plant viral pathogens were excluded. This review does not include *in silico* antiviral analyses, however, they are referred to in the discussion of the mechanisms and pharmacological effects reported in experiments and studies. The references of the included articles were also searched in order to find additional relevant studies. The plants discussed in the TPM sources and their protective mechanisms are listed in **Table 1** and the pharmacological evidence obtained from published papers are summarized in **Table 2**. **Table 3** shows a quality assessment of animal studies based on the Animal Research: Reporting of In vivo Experiments (ARRIVE) guidelines (McGrath and Lilley, 2015).

**Table 1:** Medicinal plants with possible beneficial effects in treating COVID-19 based on Traditional Persian Medicine.

Scientific name/common name	Persian names	Part	Preventing the infection of humors	Preventing the excitation of humors	Cardiotonic properties	Pulmonary tonic properties	Kidney tonic properties	Reference
*Acacia nilotica* (L.) Delile/Gum Arabic	Samgh-e-Arabi	Gum				+		(Avicenna 1025; Aghili Khorasani, 1771)
*Allium sativum* L./Garlic	Soom, Sir	Bulb	+		+		+	(Aghili Khorasani, 1771)
*Alpinia galanga* (L.) Willd., *A. officinarum* Hance/Galangal	Khoulanjān	Rhizome				+	+	(Rhazes, 960; Avicenna 1025; Aghili Khorasani, 1771)
*Aquilaria malaccensis* Lam./Agar wood	Oud-e-Hendi	Wood			+			(Avicenna 1025; Aghili Khorasani, 1771)
*Berberis vulgaris* L./Barberry	Zereshk, ambarbāris (fruit), Arghis (root)	Fruit, root	+ (root)		+ (fruit)			(Aghili Khorasani, 1771)
*Cicer arietinum* L./Pea	Nokhod	Seed				+		(Avicenna 1025; Aghili Khorasani, 1771)
*Cichorium intybus* L./Chicory	Kāsni, Hendabā	Seed, root, leaf	+	+	+		+	(Avicenna 1025; Aghili Khorasani, 1771)
*Commiphora myrrha* (Nees) Engl./Myrrh	Morr-e-Macci	oleo-gum resin	+					(Aghili Khorasani, 1771)
*Coriandrum sativum* L./Coriander	Geshniz, Kozboreh	Fruit			+			(Rhazes, 960; Aghili Khorasani, 1771)
*Crocus sativus* L./Saffron	Zaferān	Stigma			+	+	+	(Rhazes, 960; Avicenna 1025; Aghili Khorasani, 1771)
*Cydonia oblonga* Mill./Quince	Safarjal, Beh	Fruit			+			(Aghili Khorasani, 1771)
*Cymbopogon schoenanthus* (L.) Spreng./Lemon grass	Ezkher	Leaf				+	+	(Rhazes, 960; Avicenna 1025; Aghili Khorasani, 1771)
*Echium amoenum* Fisch. & C.A.Mey./Red Feathers	Lesān-al-sour, gāv zaban	Flower				+		(Avicenna 1025; Aghili Khorasani, 1771)
*Elettaria cardamomum* (L.) Maton/Cardamom	Hel	Seed			+			(Aghili Khorasani, 1771)
*Ficus carica* L./Fig	Anjeer, Tin	Fruit				+		(Avicenna 1025; Aghili Khorasani, 1771)
*Fumaria parviflora* Lam., *F. vaillantii* Loisel./Fumitory	Shātareh	Aerial parts		+				(Aghili Khorasani, 1771)
*Gentiana lutea* L./Yellow Gentian	Gentianā	Root			+			(Aghili Khorasani, 1771)
*Glycyrrhiza glabra* L./Licorice	Shirin bayan, sous	Root				+		(Rhazes, 960; Avicenna 1025; Aghili Khorasani, 1771)
*Hordeum vulgare* L./Barley	Shaeer, Jo	Seed				+		(Rhazes, 960; Avicenna 1025; Aghili Khorasani, 1771)
*Inula helenium* L./Elecampane inula	Rāsan	Root			+	+		(Rhazes, 960; Aghili Khorasani, 1771)
*Lallemantia royleana* (Benth.) Benth.	Bālangu	Seed			+			(Aghili Khorasani, 1771)
*Laurus nobilis* L./Bay laurel	Ghār, Barg-e-Bou	Leaf, fruit	+ (leaf)			+ (fruit)		(Avicenna 1025; Aghili Khorasani, 1771)
*Malus domestica* Borkh./Apple	Sib, Toffāh	Fruit			+			(Avicenna 1025; Aghili Khorasani, 1771)
*Melissa officinalis* L./Lemon balm	Bādranjbouyeh	Leaf			+			(Aghili Khorasani, 1771)
*Nymphaea alba* L./Water lily	Niloufar	Flower				+		(Avicenna 1025; Aghili Khorasani, 1771)
*Phyllanthus emblica* L./Amla	Ameleh	Fruit	+		+			(Rhazes, 960; Avicenna 1025; Aghili Khorasani, 1771)
*Pistacia lentiscus* L./Mastic	Mastaki	Oleo-gum-resin					+	(Aghili Khorasani, 1771)
*Plantago major* L., *P. lanceolata*/Plantain	Bārhang	Seed					+	(Avicenna 1025; Aghili Khorasani, 1771)
*Rheum palmatum* L./Chinese rhubarb	Reevand	Root				+		(Aghili Khorasani, 1771)
*Rosa × damascena* Herrm./Damask rose	Vard, Gol-e-Mohammadi	Flower			+	+	+	(Rhazes, 960; Avicenna 1025; Aghili Khorasani, 1771)
*Salix aegyptiaca* L./Musk willow	Beedmeshk	Flower			+		+	(Aghili Khorasani, 1771)
*Santalum album* L./Sandal wood	Sandal	Wood			+			(Avicenna 1025; Aghili Khorasani, 1771)
*Syzygium aromaticum* (L.) Merr. & L.M.Perry/Clove	Gharanfol, Mikhak	Flower bud			+		+	(Aghili Khorasani, 1771)
*Tamarindus indica* L./Tamarind	Tamr-e-Hendi	Fruit		+	+			(Aghili Khorasani, 1771)
*Trigonella foenum-graecum* L./Fenugreek	Holbeh, Shanbalileh	Seed				+		(Rhazes, 960; Avicenna 1025; Aghili Khorasani, 1771)
*Vitis vinifera* L./Grape (fresh), raisin (dried)	Enab, angour (fresh), Maveez (dried)	Fruit juice				+	+	(Avicenna 1025; Aghili Khorasani, 1771)
*Ziziphus jujuba* Mill./Jujube	Onnab	Fruit		+				(Avicenna 1025; Aghili Khorasani, 1771)

**Table 2:** Pharmacological studies on the medicinal plants predicted to be useful in SARS-CoV-2 infection based on traditional Persian medicine.

General category of therapeutic activity	Scientific name/preparation	Model/Design	Dosage and duration of treatment	Mechanisms	Reference
Antiviral activity					
	*Allium sativum*/diallyl disulfide, diallyl sulfide, alliin	*In vitro* antiviral activity against DENV-2 NGC virus in human liver & macrophage cells	10-1000 μM	↓TNF-α, IL-8, IL-10, LPO, iNOS	(Hall et al., 2017)
	***Alpinia galanga*/acetoxychavicol acetate**	*In vitro* antiviral activity against influenza virus (H1N1)	–	↓Nuclear export of viral ribonucleoprotein complex (IC_50 =_ 12.8 μM), ↓virus production (IC_50 =_ 2μM, SI=2.8)	(Watanabe et al., 2011)
	*Alpinia galanga*/acetoxychavicol acetate	*In vitro* inhibition of HIV-1 Rev	5-20 μM	↓Nuclear transport of Rev by direct binding of Cys-529 in chromosomal region maintenance-1 *via* direct binding to the nuclear export signal of Rev	(Tamura et al., 2009)
	***Alpinia officinarum*/diarylheptanoids**	*In vitro* antiviral activity against RSV, poliovirus, measles virus	–	RSV: EC_50 =_ 5-42 μg/ml, SI=0.9->6.1 Poliovirus: EC_50 =_ 3.7-44 μg/ml, SI=1-5.5 measles virus: EC_50 =_ 6-47 μg/ml, SI= 1.3-5.5	(Konno et al., 2011)
	***Alpinia officinarum*/diarylheptanoids**	*In vitro* antiviral activity against influenza A & B different subtypes, influenza A/PR/8/34-induced pulmonary infection in mouse	*In vivo*: 30, 100 mg/kg/day, 6 days	↓Viral messenger RNA & antigens, No effect on virus adsorption or invasion, EC_50_ of plaque reduction= 16-96 μM, ↑survival, ↓Body weight loss & virus titer is BALF	(Sawamura et al., 2010)
	*Crocus sativus*/aqueous extract, picrocrocin, crocin	*In vitro* antiviral activity against HIV-1	–	↓Viral replication by crocin & picrocrocin: Crocin: anti-HIV-1: IC_50_: 8 μM, SI: >187.5 Picrocrocin: anti-HIV-1: IC_50_: 5 μM, SI: >600 No significant antiviral effect by the extract	(Soleymani et al., 2018)
	*Glycyrrhiza glabra*/aqueous & alkaline extracts	*In vitro* antiviral activity against HIV-1	–	Higher anti-HIV effect with alkaline extract (EC_50 =_ 54-167 μg/ml, SI=3-9)	(Fukuchi et al., 2016)
	***Glycyrrhiza* spp./glycyrrhizin**	*In vitro* antiviral activity against influenza A H5N1-infection in human airway epithelial A549 cells	25-200 μg/ml	↓Apoptosis, viral replication, IL-6, MCP-1, CCL5, CXCL10, ROS, ↓NF-κB, JNK, & p38 activation	(Michaelis et al., 2011)
	***Glycyrrhiza* spp./glycyrrhizin**	*In vitro* antiviral activity against clinical isolates of SARS coronavirus (FFM-1 & FFM-2)	–	↓Viral replication (EC_50 =_ 300, 600 mg/l, SI=33, 67), Higher activity when added during & after virus adsorption, ↓viral antigens expression	(Cinatl et al., 2003)
	*Glycyrrhiza* spp./MeOH extract & isolated compounds	*In vitro* antiviral activity against HCV	–	IC_50_ (μg/ml): *G. uralensis* MeOH extract: 20, chloroform fraction:8, glycycoumarin:8.8, liquiritigenin:16.4, licochalcone A:2.5, glycyrin:7.2, glabridin:6.2, glycyrol:4.6, isoliquiritigenin:3.7, glycyrrhizin:180, glycyrrhizic acid monoammonium: 320, Inhibition is mostly in post-entry step	(Adianti et al., 2014)
	***Glycyrrhiza uralensis*/aqueous extract, 18β-glycyrrhetinic acid, & glycyrrhizin**	*In vitro* RSV-induced inflammation in human airway epithelial A549 & HEp-2 cells	–	18β-glycyrrhetinic acid: ↓Plaque formation (IC_50_≈71, 75 μg/ml & TI≈71, 76), Extract: ↓Plaque formation (IC_50_≈4 μg/ml & TI≈27), ↓viral attachment & internalization, ↑IFNβ	(Feng Yeh et al., 2013)
	*Phyllanthus emblica*/aqueous & MeOH extract	*In vitro* HIV-RT inhibition assay	–	IC_50 =_ 9 μg/ml for aqueous & 10 μg/ml for MeOH extracts. putranjivain A from MeOH extract showed IC_50 =_ 3.9 μM	(el-Mekkawy et al., 1995)
	*Rheum palmatum* & *Rheum officinale*/hydromethanolic extract & isolated compounds	*In vitro* antiviral activity against HIV-1	–	IC_50_: RNase H: *R. palmatum*=0.9 μg/ml, *R. officinale*=0.25 μg/ml, sennoside A=1.9 μM, sennoside B=2.1 μM RDDP: sennoside A=5.3 μM, sennoside B=2.3 μM, Integrase: sennoside A=3.8 μM, sennoside B=87 μM, ↓viral replication	(Esposito et al., 2016)
	*Rheum palmatum*/EtOH extract	*In vitro* antiviral activity against CVB_3_, CVB_3_-induced infection in mouse	*In vitro*: 2-10 μg/ml, *In vivo*: 0.18-0.5 mg/kg/day, i.p., 5 days	*In vitro*: ↓viral replication (IC_50 =_ 4 μg/ml, SI=10), *In vivo*: ↑survival, ↓viral titer	(Xiong et al., 2012)
	*Rheum* spp./emodin	*In vitro* antiviral activity against EV71	29.6 μM	↓Viral replication, maturation, & virulence, Lower effect of viral protein expression, ↓cell cycle arrest at S phase	(Zhong et al., 2017)
	*Rosa damascena*/MeOH & aqueous extracts, purified flavonoids	*In vitro* antiviral activity against HIV	–	↓Infectivity (EC_50 =_ 4- >250 μg/ml, SI=5- >100), ↓gp 120 binding to CD4, viral protease	(Mahmood et al., 1996)
	*Syzygium aromaticum*/aqueous & MeOH extracts	*In vitro* inhibition of HCV protease	100 μg/ml	76% % 90% inhibition by the MeOH & aqueous extracts, IC_50 =_ 33 μg/ml for aqueous extract	(Hussein et al., 2000)
	***Syzygium aromaticum*/eugenol from essential oil**	*In vitro* CPE inhibitory assay against influenza A virus	5 μg/ml	↓Virus-induced autophagy & cell death, virus replication, ↓ROS, NO, LPO, IL-1, IL-6, IL-8, TNF-α, ↓activation of ERK1/2, p38MAPK, & IKK/NF-kB pathways but not JNK1, ↑GSH, GR, SOD	(Dai et al., 2013)
	*Vitis vinifera*/extract	*In vitro* antiviral activity against HCV	2.5-20 μg/ml	↓HCV replication, COX-2, NF-κB & MAPK/ERK/JNK signaling, Synergistic effect with conventional anti-HCV drugs	(Chen et al., 2016)
	***Vitis vinifera*/proanthocyanidin extract**	*In vitro* RSV-induced inflammation in human airway epithelial A549 cells	5, 10 μg/ml	↓Viral replication, viral nucleoprotein & fusion protein, ↓MUC1, MUC2, MUC5B, MUC8 expression & mucin synthesis, Suppression of AP-1 & NF-κB *via* p38 MAPKs/JNK	(Lee et al., 2017)
	***Zizyphus jujuba*/betulinic acid**	*In vitro* antiviral activity against influenza A/PR/8 virus, Antiviral activity in mouse infected with influenza A/PR/8 virus	*In vitro*: 0.4-50 μg/ml *In vivo*: 10 mg/kg/day, i.p., 7 days	*In vitro*: ↓viral infection, *In vivo*: ↓pulmonary necrosis, inflammation, edema, leukocytes infiltration, ↓IFNγ, No effect on TNF-α, IL-1β, & virus replication	(Hong et al., 2015)
Cardioprotective activity	*Allium sativum*/allicin	DOX-induced cardiotoxicity in rat	20 mg/kg/day, p.o., 14 days	↑CAT, SOD, Gpx, ↓LPO, LDH, CK-MB, NO, TNF-α, IL-1β, COX-2, Casp-3	(Abdel-Daim et al., 2017)
	*Allium sativum*/alliin	I/R-induced cardiotoxicity in mouse	100 mg/day, i.p., two doses	↓Cardiomyocyte apoptosis & infarct size, ↑autophagic flux, LC3II/LC3I, beclin-1, Atg9b	(Zhao et al., 2019)
	*Allium sativum*/diallyl trisulfide	*In vitro* cytoprotection in H9c2 murine cardiocyte, I/R-induced cardiotoxicity in STZ-induced diabetic rat	*In vitro*: 10 μM, *In vivo*: 20 mg/kg/day, p.o., 3 days	*In vitro* & *in vivo*: ↓Apoptosis, ↑AMPK-mediated AKT/GSK-3β/HIF-1α activation	(Yu et al., 2017)
	*Allium sativum*/homogenate	Fructose-induced cardiotoxicity in diabetic rat	250 mg/kg/day, p.o., 8 weeks	↓NF-κB, ROS, LPO, NO, ↑CAT, GSH, Gpx, SOD, Modulation of PI3K/AKT/Nrf2-Keap1 pathway	(Padiya et al., 2014)
	*Alpinia galanga*/Cardamonin	DOX-induced cardiotoxicity in mouse	20-80 mg/kg/day, p.o., 28 days	Improvement of cardiac function, ↑Nrf-2 signaling, HO-1, NQO-1, GCLM, SOD, GSH, CAT, ↓LPO, ROS, & apoptosis	(Qi et al., 2020)
	*Crocus sativus*/aqueous extract	ISO-induced cardiotoxicity in rat	200-800 mg/kg/day, p.o., 28 days	Improvement of hemodynamic function of heart, ↑Cardiac SOD, CAT, GSH, ↓LPO, LDH & CK-MB leakage	(Sachdeva et al., 2012)
	*Crocus sativus*/aqueous extract, safranal	ISO-induced cardiotoxicity in rat	20-160 mg/kg/day of extract or 0.025-0.075 of safranal, i.p., 9 days	↓LDH, CK-MB, LPO, Improvement of myocardium morphological changes	(Mehdizadeh et al., 2013)
	*Crocus sativus*/crocin	*In vitro* LPS-induced cardiotoxicity in H9c2 murine cardiocyte	10-40 μM	↑Viability, thiol content ↓TNF-α, PGE2, IL-1β, and IL-6, NO, ↓TNF-α, COX-2, IL-1β, IL-6, & iNOS gene expression	(Rahim et al., 2019)
	*Crocus sativus*/crocin	DOX-induced cardiotoxicity in rat	20, 40 mg/kg/day, i.p., 20 days	Improvement of heart function, ECG, & histopathological damages	(Razmaraii et al., 2016)
	*Crocus sativus*/hydromethanolic extract	*In vitro* I/R+DOX-induced toxicity in H9c2 murine cardiocyte	10 μg/ml	↑Viability, α-actinine, troponine C & MLC, AKT/P70S6K & ERK1/2 activity, ↓Casp-3, LDH, mitochondrial dysfunction	(Chahine et al., 2016)
	*Glycyrrhiza glabra*/aqueous extract	*In vitro* DOX-induced toxicity in H9c2 murine cardiocyte	20-200 μg/ml	↓ROS, DNA damage, mitochondrial dysfunction, ↑membrane integrity, actin stability, SIRT-1, PPARγ & PPARα	(Upadhyay et al., 2020)
	*Glycyrrhiza glabra*/hydroalcoholic extract	I/R-induced cardiotoxicity in rat	400 mg/kg, p.o., 30 days	↑SOD, Gpx, CAT, GSH, Restoration of LDH, CK-MB, & hemodynamic cardiac function, ↓LPO	(Ojha et al., 2013)
	*Glycyrrhiza* spp./glycyrrhizic acid	ISO-induced cardiotoxicity in rat	10, 20 mg/kg, p.o., 2 days	Modulation of ECG & morphology, ↓CK-MB, LDH, Reversible inhibition of L-type Ca^2+^ channels in isolated rat cardiomyocytes (EC_50 =_ 145.54 μg/mL)	(Li et al., 2019)
	*Phyllanthus emblica*/aqueous extract	I/R-induced cardiotoxicity in rat	100 mg/kg/day, p.o., 30 days	Upregulation of PI3K/Akt/GSK3β/β-catenin & Bcl-2, ↑eNOS phosphorylation, ↓myocardiocyte apoptosis,	(Thirunavukkarasu et al., 2015)
	*Phyllanthus emblica*/emblicanin-A & -B enriched fraction	I/R-induced cardiotoxicity in rat	100 & 200 mg/kg/day, p.o., 14 days	↑Cardiac SOD, CAT, Gpx, ↓LPO	(Bhattacharya et al., 2002)
	*Phyllanthus emblica*/hydroalcoholic extract	ISO-induced cardiotoxicity in rat	100-500 mg/kg/day, p.o., 30 days	Restoration of hemodynamic parameters & cardiac function, ↑Cardiac SOD, CAT, Gpx, GSH, ↓LPO, ↓LDH & CK-MB leakage from myocardium	(Ojha et al., 2012)
	*Phyllanthus emblica*/juice	STZ-induced diabetic myocardial dysfunction in rat	1 ml/kg/day, p.o., 8 weeks	Restoration of hemodynamic parameters, ↓LDH & CK-MB	(Patel and Goyal, 2011)
	*Rheum officinale*/emodin	Isolated perfused beating rabbit atria	10-100 μM	↑Atrial natriuretic peptide, ↓atrial pulse pressure & stroke volume, Involvement of L-type Ca^2+^ & K^+^ channel, No change in muscarinic system	(Zhou et al., 2014)
	*Rheum palmatum*/chrysophanol	*In vitro* DOX-induced toxicity in H9c2 murine cardiocyte, DOX-induced cardiotoxicity in rat	*In vitro*: 1-20 μM, *In vivo*: 5-40 mg/kg/day, p.o., 7 days	*In vitro*: ↓apoptosis, cleavage & activation of PARP1, Casp-3, cytochrome c release from mitochondria to cytoplasm, ↑Bcl-2/Bax, *In vivo*: modulation of ECG, ↓fibrosis, apoptosis, mitochondrial damage	(Lu et al., 2019)
	*Rheum palmatum*/rhein	I/R-induced cardiotoxicity in H9c2 murine cardiocyte	1 μg/ml	↓Apoptosis, ROS, p-P38, ↑AKT & GSK3β phosphorylation	(Liu et al., 2018)
	*Rosa damascena*/hydroethanolic extract	Isolated guinea pig heart un-treated or pretreated with propranolol, methacholine, & diltiazem	0.1-1 mg%	↑Heart rate & contractility, Higher activity of the extract vs. isoprenaline	(Boskabady et al., 2013)
	*Trigonella foenum-graecum*/digoxigenin-3-O-rutin	ISO-induced cardiotoxicity in rat	2.5-10 mg/kg/day, p.o., 10 days	↓CK-MB, Cr, LDH, AST, ALT, LPO, ↑GSH, Gpx, GST, SOD, CAT, Na^+^-K^+^-ATPase	(Panda and Kar, 2010)
	*Trigonella foenum-graecum*/polysaccharide	*In vitro* cytoprotection in H9c2 murine cardiocyte, Thiamethoxam-induced cardiotoxicity in rat	0.01-1 mg/ml, 100 & 200 mg/kg/day, p.o., 30 days	*In vitro*: ↓H9c2 necrosis & apoptosis, *In vivo*: ↓LDH, CPK, AST, troponin-T, LDL, TAG, LPO & protein oxidation, ↑GSH & NPSH	(Feki et al., 2019)
	*Trigonella foenum-graecum*/powder	ISO+ hypercholesterolemic diet-induced cardiotoxicity in rat	10% of diet, p.o., 8 weeks	↓LDH, CK-MB, AST, ALT, Improvement of the lipid profile & histology of the heart muscle	(Mukthamba and Srinivasan, 2015)
	*Trigonella foenum-graecum*/seed powder	STZ-induced cardiotoxicity in diabetic rats	9 g/kg/day, p.o., 30 days	↑Activity of cardiac SOD, CAT, GSH, GST, ↓LPO	(Tripathi and Chandra, 2009)
	*Trigonella foenum-graecum*/seed powder	STZ-induced cardiotoxicity in diabetic rats	10% of the diet weight, 6 weeks	↓RAS activity, type IV collagen, fibronectin, Bax, 4-hydroxynonenal, iNOS, nitrate/nitrite, ↑PUFA/SFA ratio	(Pradeep and Srinivasan, 2018)
	*Trigonella foenum-graecum*/trigonelline	ISO-induced cardiotoxicity in rat	20-80 mg/kg/day, p.o., 20 days	↓Infarction area, ↓CK-MB, LDH, LPO, ALT, ↓Hsp27 & αB crystallin, ↑GSH, Gpx, GST, SOD, CAT	(Panda et al., 2013)
	*Vitis vinifera*/proanthocyanidin extract	DOX-induced cardiotoxicity in rat	70 mg/kg, p.o., 10 days	Modulation of ECG, ↓CK-MB, LDH, LPO, ↑SOD, CAT	(Ammar el et al., 2013)
	*Vitis vinifera*/proanthocyanidin extract	*In vitro* I/R-induced cardiotoxicity in H9c2 murine cardiocyte	50-200 μg/ml	↑Viability, ↓LDH, GRP78, CHOP, phosphorylated PERK, eIF2α, endoplasmic reticulum stress‑induced apoptosis	(Wang X. et al., 2017)
	*Zizyphus jujuba*/Jujuboside A	ISO-induced cardiotoxicity in H9c2 murine cardiocytes	5-20 μM	↑Viability, phosphorylation of PI3K, Akt, & mTOR, ↓LC3-II/I	(Han et al., 2016)
	*Zizyphus jujuba*/polyphenols	ISO-induced cardiotoxicity in rat	300 mg/kg/day, p.o., 5, 10 days	↓LPO, Ca^2+^ & Mg^2+^-ATPase activity, ↑SOD, Gpx, Na^+^K^+^-ATPase, LDH & CK activity, Modulation of ECG	(Cheng et al., 2012)
Immunomodulatory activity	*Allium sativum*/aged extract	Randomized, double-blind, placebo-controlled trial in healthy individuals	2.6 g/day, p.o., 90 days	↑Proliferation of γδ-T cells & NK cells, ↓severity of cold & flu symptoms	(Nantz et al., 2012)
	*Allium sativum*/essential oil & organosulfur compounds	*In vitro* human neutrophils	–	↑Ca^2+^ flux (EC_50 =_ 9-22 μM), ↑ROS production *via* PI3K activation, CREB, ERK1/2, & GSK-3α/β phosphorylation (200-500 μM of 1,3-dithiane)	(Schepetkin et al., 2019)
	*Allium sativum*/polysaccharides	*In vitro* immunostimulation RAW 264.7 macrophages	5-200 μg/ml	↑Proliferation, phagocytosis, secretion of NO, IL-6, IL-10, TNF-α, IFNγ, Higher activity by the fresh sample	(Li et al., 2017)
	*Alpinia galanga*/acetoxychavicol acetate	*In vitro* LPS-activated murine macrophage	0.3-10 μM	↓IFNβ mRNA expression, NF-κB activation, NO production *via* TLR-3	(Ando et al., 2005)
	*Alpinia galanga*/galangin	*In vitro* LPS-activated RAW 264.7 macrophages	12.5- 50 μM	↓iNOS, NO, IL-6, IL-1β, ERK & NF-κB-p65 phosphorylation	(Jung et al., 2014)
	*Cichorium intybus*/EtOH extract	EtOH-induced immunotoxicity in mouse	300 mg/kg/day, p.o., 28 days	↑Circulating leukocytes, splenic plaque forming cells, hemagglutination titers to SRBC, secondary IgG response to bovine serum albumin, phagocytes activity, NK cells, IFNγ, delayed-type hypersensitivity	(Kim et al., 2002)
	*Cichorium intybus*/powder	Innate immune response in growing piglets	4% as dietary supplement, p.o., 21 days	↓Apolipoprotein C-II complement component C6, CRP, CD14 antigen, C4b binding protein α & β chains, fibrinogen	(Lepczynski et al., 2015)
	*Crocus sativus*/hydroethanolic extract	Non-stimulated & phytohemagglutinin-stimulated lymphocytes	50-500 μg/ml	Stimulated cells: ↓IFNγ, IL-10, Non-stimulated cells: ↑IFNγ, IL-4 ↑Th1/Th2 balance	(Boskabady et al., 2011)
	*Crocus sativus*/powder	Randomized double‐blind placebo‐controlled in healthy men	100 mg, p.o., 6 weeks	Week 3: ↑IgG, monocyte percentage, ↓IgM, basophil percentage, Week 6: all results returned to baseline	(Kianbakht and Ghazavi, 2011)
	*Glycyrrhiza* spp./glycyrrhizin	*In vitro* LPS-induced inflammation in RAW 264.7 macrophage, LPS-induced endotoxemia in mouse	*In vitro*: 0.5-2 μM, *In vivo*: 200 mg/kg, i.p., single dose	↑HO-1, nuclear translocation of Nrf2, ↓HMGB1, iNOS, Involvement of p38, but not ERK or JNK	(Kim et al., 2015)
	*Phyllanthus emblica*/EtOH extract	*In vitro* Chromium (VI)-induced immunosuppression in rat lymphocyte	10-1000 μg/ml	Restoration of IL-2 & IFNγ production ↑cell survival, GSH, Gpx, ↓LDH leakage, ROS, LPO, DNA fragmentation	(Sai Ram et al., 2002)
	*Phyllanthus emblica*/hydroalcoholic extract	*In vitro* Chromium (VI)-induced immunosuppression in J-774 macrophage	250 μg/ml	Restoration of phagocytosis & IFNγ production, ↑cell survival, Gpx & GSH, ↓ROS	(Sai Ram et al., 2003)
	*Syzygium aromaticum*/biflorin	*In vitro* LPS-induced inflammation in RAW 264.7 macrophage, LPS-induced endotoxemia in mouse	5 & 10 mg/kg, i.p., single dose	↓iNOS, NO, COX-2, PG-E2, TNF-α, IL-6, p-STAT1, p-p38	(Lee et al., 2016)
	*Syzygium aromaticum*/essential oil	Cyclophosphamide-induced immunosuppression in mouse under SRBC challenge	100-400 mg/kg/day, p.o., 7 days	↑WBC, Stimulation of both humoral & cell-mediated immunity	(Carrasco et al., 2009)
	*Syzygium aromaticum*/hydromethanolic extract & eugenol	*In vitro* LPS-induced inflammation in murine peritoneal macrophage	5-100 μg/well	↓IL-1β by the extract, ↓IL-6 & IL-10 by the extract & eugenol	(Bachiega et al., 2012)
	*Trigonella foenum-graecum*/powder	Burn wound induced in cyclophosphamide-immunosuppressed rat	0.5 & 1 g/kg/day, p.o., 28 days	↓Neutropenia & lymphopenia, ↑weight & cellularity of thymus, spleen, bone marrow, ↑γ-globulin, delayed-type hypersensitivity response, & burn wound healing rate	(Ramadan et al., 2011)
	*Vitis vinifera*/proanthocyanidin extract	Aflatoxin-induced immunotoxicity in mouse	50, 100 mg/kg/day, p.o., 3 weeks	↓Weight loss, ↓splenic LPO, IL−1β, IL-6, TNF-α, IFNγ, ↑CAT, GSH, Gpx, SOD	(Long et al., 2016)
	*Zizyphus jujuba* cv. *Huizao*/acidic polysaccharides	Immunomodulatory effect in healthy mouse	50-200 mg/kg/day, p.o., 7 days	↑Spleen & thymus indices, hemagglutination titers to SRBC, delayed-type hypersensitivity, phagocytes activity	(Zou et al., 2018)
Lung protective activity	*Allium sativum*/aqueous extract	Lambda-cyhalothrin-induced pulmonary damage in rat	100 mg/kg/day, i.p., 21 days	↓Cough, nasal discharge, alveolitis, lung inflammation & hyperplasia	(Mohi El-Din et al., 2014)
	*Allium sativum*/S-allyl-L-cysteine	Bleomycin-induced pulmonary toxicity in mouse	5, 10 mg/kg, i.p., single dose	↓Pulmonary fibrosis *via* α-SMA, TNF-α, fibronectin, collagen I & III, leukocytes infiltration into BALF, iNOS, AKT & NF-κB p65 phosphorylation	(Nie et al., 2019)
	*Allium sativum*/S-allylmercaptocysteine	LPS-induced lung inflammation in mouse	10-60 mg/kg, p.o., single dose	↓Lung edema, MPO, TNF-α, IL-1β, IL-6, iNOS, COX-2, NF-κB, LPO, ↑GSH, SOD, HO-1, Modulation of Keap1/Nrf2 pathways	(Mo et al., 2020)
	*Alpinia galanga*/acetoxychavicol acetate	OVA-induced airway inflammation in mouse	25, 50 mg/kg/day, i.p., 5 days	↓Leukocyte infiltration in BALF, airway hyperresponsiveness, goblet cells hyperplasia, ↓anti-OVA IgG, IL-4, IL-13, IL-12α, IFNγ	(Seo et al., 2013)
	*Alpinia galanga*/galangin	LPS-induced airway inflammation in mouse	1.5, 15 mg/kg/day, i.p., single dose	↑HO-1 & oxygenation, ↓lung edema, NF-κB, MPO, IL-6, TNF-α	(Shu et al., 2014)
	*Alpinia galanga*/galangin	*In vitro* TNF-α-induced inflammation in normal human airway smooth muscle cells, OVA-induced airway inflammation in mouse	10 μM *in vitro* 5, 15 mg/kg/day, i.p., 3 days	*In vitro*: ↓MCP-1, nuclear translocation of p65, eotaxin, CXCL10, and VCAM-1, *In vivo*: ↓leukocyte infiltration in BALF, airway hyperresponsiveness, goblet cells hyperplasia, ↓anti-OVA IgG, IL-4, IL-5, IL-13, iNOS, VCAM-1, NF-κB-related inflammation	(Zha et al., 2013)
	*Crocus sativus*/crocin	*In vitro* cytoprotection in HUVEC cells, LPS-induced lung inflammation in mouse	*In vitro*: 20 μM, *In vivo*: 15-60 mg/kg, i.p., 7 days	*In vitro* & *in vivo*: ↓NF-κB & MAPK activity, MMP-9, heparanase, ↑Pulmonary vascular permeability	(Zhang et al., 2020b)
	*Crocus sativus*/crocin	Bleomycin-induced pulmonary toxicity in rat	20 mg/kg/day, p.o., 5 weeks	↓Pulmonary inflammation, fibrosis, leukocytes infiltration into BALF ↓LDH, LPO, NO, TNF-α, TGF-β1, TLR-4, IL-10, ↑SOD, GSH, TAC, HO-1, Nrf2	(Zaghloul et al., 2019)
	*Crocus sativus*/powder	Randomized, triple-blind, placebo-controlled trial in patients with mild & moderate persistent asthma	100 mg/day, p.o., 8 weeks	Improvement of spirometry parameters, ↓CRP, anti-Hsp70 antibody	(Hosseini et al., 2018)
	*Crocus sativus*/safranal	OVA-induced airway inflammation in guinea pigs	4-16 μg/ml of drinking water	↓NO, nitrite, IL-4, tracheal response to methacholine & OVA, ↑IFNγ/IL-4	(Boskabady et al., 2014)
	*Glycyrrhiza glabra*/glycyrrhizic acid	OVA-induced airway inflammation in mouse	10-40 mg/kg/day, p.o., 30 days	↑Regulatory T cells, IFNγ, Foxp3 protein, ↓leukocytes infiltration into BALF, IL-4, IL-5, IL-13, OVA-specific IgE	(Ma et al., 2013)
	*Glycyrrhiza glabra*/glycyrrhizin	*In vitro* TGF-α-induced mucus production in NCI-H292 cells, LPS & IL-4-induced airway inflammation in mouse	*In vitro*: 10-1000 μM, *In vivo*: 15-135 mg/kg, s.c., 6 days	*In vitro*: ↓MUC5AC protein and mRNA expression *In vivo*: ↓Goblet cell hyperplasia & MUC5AC mRNA expression	(Nishimoto et al., 2010)
	*Glycyrrhiza glabra*/aqueous extract	Randomized, double-blind, placebo-controlled trial in 235 patients with postoperative sore throat & postextubation coughing	0.5 g/30 ml, as gargle,	↓Sore throat & incidence of coughing	(Ruetzler et al., 2013)
	*Glycyrrhiza uralensis*/glycyrrhizic acid	*In vitro* LPS-induced inflammation in RAW 264.7 macrophage, LPS-induced lung inflammation in mouse	*In vitro*: 100 μg/ml, *In vivo*: 200 mg/kg, i.p., single dose	↑viability, LC3-II/I and Beclin-1, autophagy *via* PI3K/AKT/mTOR pathway, ↓TNF-α, IL-1β, HMGB1	(Qu et al., 2019)
	*Phyllanthus emblica*/EtOH extract & pyrogallol	*In vitro* cytoprotective effects against *P. aeruginosa* damage in IB3-1 bronchial epithelial cells	500 μg/ml of the extract, 2, 20, 200 μM pyrogallol	↓IL-6, IL-8, GRO-α, GRO-γ, & ICAM-1, No effect on bacterial adhesion	(Nicolis et al., 2008)
	*Rheum officinale*/emodin	*In vitro* TGF-β1-induced toxicity in human embryo lung fibroblasts, Bleomycin-induced pulmonary toxicity in rat	*In vitro*: 15-60 μM, *In vivo*:10-40 mg/kg/day, p.o., 21 days	*In vitro*: ↓α-SMA, collagen IV, fibronectin, Smad2/3 & STAT3 activation *In vivo*:↓Pulmonary edema & fibrosis, TNF-α, IL-6, TGF-β1, α-SMA, HSP-47	(Guan et al., 2016)
	*Rheum palmatum*/aqueous extract	Randomized, controlled trial in patients with acute respiratory distress syndrome treated with the extract+ conventional drugs or only conventional drugs	10 g/30 ml, TDS, p.o., 7 days	↑Oxygenation, ↓Extravascular lung water index, pulmonary vascular permeability index	(He et al., 2017)
	*Rheum palmatum*/chrysophanol	*In vitro* TNF-α-induced toxicity in human pulmonary epithelial BEAS‐2B cells, OVA-induced airway inflammation in mouse	*In vitro*: 2, 20 μM, *In vivo*: 0.1-10 mg/kg/day, i.p., 4 days	*In vitro*: inhibition of NF‐κB pathway, *In vivo*: ↓IL-4, IL-5, IL-13, TNF-α, iNOS, pulmonary α‐SMA expression & airway remodeling, NF‐κB p65 activation & nuclear translocation, ↓autophagy	(Song et al., 2019)
	*Rheum palmatum*/rhein	RSV-induced pulmonary damage in mouse	30-120 mg/kg/day, p.o., 5 days	Improvement of lung index, ↓IL-1β, IL-6, IL-18, IL-33, TNF-α, ↓NF-κB-dependent NLRP3 inflammasome activation	(Shen et al., 2019)
	*Rosa damascena*/EtOH extract & essential oil	*In vitro* KCl, methacholine, & methacholine + propranolol + chlorpheniramine-induced contraction in tracheal chains of guinea pig	0.25-1%	Relaxation in KCl & methacholine-induced tracheal contraction, Higher activity by the essential oil vs. theophylline	(Boskabady et al., 2006)
	*Syzygium aromaticum*/aqueous extract	*In vitro* cytoprotective effect on human neutrophil, LPS-induced lung inflammation in mouse	200 mg/kg, two doses, i.p.	↓MPO in neutrophils, ↓neutrophil count, protein leakage in alveoli, MMP-2 & -9 activity	(Chniguir et al., 2019)
	*Syzygium aromaticum*/eugenol	LPS-induced lung inflammation in mouse	160 mg/kg, i.p.	Improvement of lung function, ↓alveolar collapse, collagen fibers, & neutrophil influx, ↓NF-κB activation & TNF-α	(Magalhaes et al., 2010)
	*Trigonella foenum-graecum*/hot water extract as syrup	Randomized controlled trial in patients with mild asthma	10 ml, BD, 4 weeks	Improvement of spirometry parameters & quality of life, ↓IL-4	(Emtiazy et al., 2018)
	*Trigonella foenum-graecum*/hydroalcoholic extract	Bleomycin-induced pulmonary toxicity in rat	5-40 mg/kg/day, p.o., 28 days	Improvement of lung function & hematological parameters, ↓BALF differential cells, ↑peripheral blood oxygen content, SOD, GSH, CAT, Bcl-2, TAC, ↓NO, HO-1, LPO, Nrf-2, IL-1β, IL-6, IL-8, TNF-α, hydroxytriptamine, hydroxyproline, histamine, TGF-β, LDH, ALP, collagen-1, ET-1, NF-κB, VEGF, Smad-3, Bax, Casp-3	(Kandhare et al., 2015)
	*Vitis vinifera*/polyphenolic extract	Bleomycin-induced pulmonary toxicity in mouse	50, 100 mg/kg/day, p.o., 21 days	↓Leukocytes infiltration in BALF, hydroxyproline, TGF-β1, MMP-9, collagen 1-α1, fibronectin-1, ↑E-cadherin	(Liu et al., 2017)
	*Vitis vinifera*/proanthocyanidin extract	*In vitro* RSV-induced inflammation in human airway epithelial A549 cells	5, 10 μg/ml	↓IL−1β, IL−6, IL−8 mRNA & protein expression	(Kim et al., 2019)
	*Vitis vinifera*/proanthocyanidin extract	*In vitro* As-induced toxicity in human lung epithelial BEAS-2B cells, As-induced pulmonary toxicity in mouse	*In vitro*: 25, 50 mg/ml, *In vivo*: 400 mg/kg/day, p.o., 5 weeks	*In vitro* & *in vivo*: ↓Apoptosis, LPO, ROS, IL−1β, IL−6, CRP, TNF-α, NF-κB activation, ↑IL-10, ↓lung inflammation & edema *in vivo*	(Hu et al., 2019)
	*Vitis vinifera*/proanthocyanidin extract	Pb-induced pulmonary toxicity in rat	200 mg/kg/day, p.o., 5 weeks	↑AMPK/Nrf2/p62 signaling activation, ↑GSH, SOD, γ-GCS, Bcl-2, NQO1, ↓Pb pulmonary concentration, apoptosis, LPO, Bax, p53, TNF-α, NF-κB nuclear translocation, HO-1	(Lu et al., 2018)
	*Vitis vinifera*/proanthocyanidin extract	Carrageenan-induced pulmonary inflammation in mouse	25-100 mg/kg, p.o., single dose	↓IL-17A & GITR expressing cells, ↓IL-17A, IL-1β, IL-2, IL-6, IL-12, IFNγ, ICAM-1, TNF-α, MCP-1, ↑TGF-β1, IL-4, IL5, IL-10	(Ahmad et al., 2014)
Nephroprotective activity	*Allium sativum*/diallyl trisulfide	As-induced nephrotoxicity in rat	80 mg/kg/day, p.o., 28 days	↓BUN, Cr, ↓renal As concentration, membranes bound ATPases, Bax, Cyt C, Nox2, p47phox & Nox4, TNF-α, IL-1β, IL-6, iNOS, NF-κB, Casp-3, ↑renal SOD, CAT, GST, Gpx, GR, G6PD, GSH, TSH, vitamin C & E, ↑Akt, PI3K & their phosphorylated form, Bcl-2	(Miltonprabu et al., 2017)
	*Allium sativum*/S-allylmercaptocysteine	*In vitro* cisplatin-induced cytotoxicity in human kidney HK-2 cells, Cisplatin-induced nephrotoxicity in rat	*In vitro*: 50-100 μM, *In vivo*: 10-30 mg/kg/day, i.p., 20 days	*In vitro*: ↓apoptosis, cleaved PARP, p53, ↑Bcl-2, *In vivo*: ↓tubular damage, NF-κB, LPO, TNF-α, IL-1β, TGF-β1, COX-2, ↑Nrf2, NQO1, CAT, SOD, GSH	(Zhu et al., 2017)
	*Alpinia galanga*/galangin	High-fructose diet-induced nephrotoxicity in rat	50-200 μg/kg/day, p.o., 60 days	↓LPO, Micro-albuminuria & tubular glomerular damage, ↑renal & plasma SOD, CAT, Gpx, GSH, vitamin C & E	(Sivakumar et al., 2010)
	*Cichorium intybus*/Aqueous extract	*In vitro* cytoprotection in HCK cells, Adenine + yeast-induced chronic kidney disease in rat	*In vitro*: 100-400 μg/ml, *In vivo*: 6.6, 13.2 g/kg/day, p.o., 5 weeks	*In vitro*: ↓transmembrane transport of uric acid, *In vivo*: ↓serum uric acid & Cr, microalbuminuria, GLUT-9 protein expression,	(Jin et al., 2018)
	*Cichorium intybus*/Aqueous extract	STZ-induced diabetic nephropathy in rat	125 mg/kg/day, i.p., 21 days	↓serum uric acid & Cr, microalbuminuria, & renal morphological damage	(Pourfarjam et al., 2017)
	*Crocus sativus*/aqueous extract	EtOH-induced nephrotoxicity in rat	40-160 mg/kg/day, p.o., 4 weeks	↓Renal LPO, TNF-α, IL-6, Casp-3, Casp-8, Casp-9, Bax/Bcl2, ↑GSH	(Rezaee-Khorasany et al., 2019)
	*Crocus sativus*/crocin	Tartrazine-induced nephrotoxicity in rat	50 mg/kg/day, p.o., 21 days	↓BUN, Cr, renal LPO, ↑GSH, TAC, SOD, CAT	(Erdemli et al., 2017)
	*Crocus sativus*/crocin	STZ-induced diabetic nephropathy in rat	20 mg/kg/day, p.o., 21 days	↓Tubular necrosis, inflammation, & desquamation, ↓BUN, Cr, LPO, xanthine oxidase activity ↑GSH	(Altinoz et al., 2015)
	*Glycyrrhiza* spp./glycyrrhizic acid	*In vitro* LPS-induced toxicity in rat mesangial cells, LPS-induced nephrotoxicity in rat	*In vitro*: 50, 100 μM, *In vivo*: 25, 50 mg/kg, i.p., single dose	*In vitro*: ↓apoptosis, Casp-3, iNOS, NO, COX-2, PGE2, ROS, NF-κB activation, ↑Bcl-2/Bax, HO-1, *In vivo*: ↓BUN, Cr, TNF-α, MCP-1, ICAM-1, VCAM-1	(Zhao et al., 2016)
	*Phyllanthus emblica*/emblicanin-A & -B enriched extract	Cisplatin-induced nephrotoxicity is rat	150-600 mg/kg/day, p.o., 10 days	↑Renal CAT, GSH, SOD, ↓Inflammation & apoptosis, ↓LPO, MAPK phosphorylation	(Malik et al., 2016)
	*Rheum officinale*/different extracts	Adenine-induced chronic kidney disease in rat	200-800 mg/kg/day, p.o., 6 weeks	↓Renal α-SMA, collagen-I & collagen-III, ↓BUN, Cr, TGF-β1, TGF-β receptor I & II, Smad-2, Smad-3, Smad4, vimentin ↑Smad7, E-cadherin	(Zhang et al., 2018)
	*Rheum palmatum*/aqueous extract, rhein	*In vitro* Hank’s balanced salt solution-induced autophagy in NRK-52E normal rat kidney cells, Adenine-induced chronic kidney disease in rat	*In vitro*: 1, 10 μM, *In vivo*: 1 g/kg/day, p.o., 3 weeks	*In vitro*: ↓autophagy *via* AMPK-dependent mTOR signaling pathway, Erk & p38 MAPKs by rhein *In vivo*: ↓Renal fibrosis, collagen-1, fibtonectin, LC3 conversion by the extract	(Tu et al., 2017)
	*Syzygium aromaticum*/aqueous extract	Infectious pyelonephritis in rat	500 mg/kg/day, p.o., 28 days	↓Leukocyte count, Normalization of histomorphological changes	(Nassan et al., 2015)
	*Trigonella foenum-graecum*/seed powder	STZ-induced diabetic nephropathy in rat	10% of the diet weight, 6 weeks	↓Renal Glut-1 & -2, ACE, iNOS, & NO, ↓renal AST, ALT, alkaline & acid phosphatases, Na^+^, K^+^, ouabain-sensitive, Mg^2+^ATPase, & Ca^2+^ATPase, fructose 1,6-diphosphatase, G6 Pase, LDH activity, ↑renal hexokinase & G6PD activity, ↓urinary excretion of proteins, ↓renal polyol pathway enzyme activity, ↓podocyte damage & morphological changes	(Pradeep et al., 2019)
	*Vitis vinifera*/proanthocyanidin B2	*In vitro* glucosamine-induced nephrotoxicity in rat mesangial cells	2.5, 10 μg/ml	↓Apoptosis & mitochondrial dysfunction, ↑Gpx, SOD, PGC-1α, SIRT1, AMPK, NRF1,	(Bao et al., 2015)
	*Vitis vinifera*/proanthocyanidin extract	STZ-induced diabetic nephropathy in rat	250 mg/kg/day, p.o., 16 weeks	No significant change in BUN & Cr, ↓renal index, urinary albumin, endoplasmic reticulum stress‑induced apoptosis *via* Casp‑12	(Gao et al., 2018)
	*Vitis vinifera*/proanthocyanidin extract	As-induced nephrotoxicity in mouse	400 mg/kg/day, p.o., 5 weeks	↓NF-κB activation, IL−1β, IL−6, CRP, TNF-α, ↑IL-10	(Wang C. et al., 2017)
	*Vitis vinifera*/proanthocyanidin extract	Diatrizoate-induced nephrotoxicity in rat	100 mg/kg/day, p.o., 8 days	↓BUN, Cr, Casp-1 & -3, calpain-1, iNOS, eNOS, Better effect than N-acetylcysteine	(Ulusoy et al., 2014)

SOD, superoxide dismutase; CAT, catalase; Gpx, glutathione peroxidase; GSH, glutathione; GR, glutathione reductase; GST, glutathione-S-transferase; LPO, lipid peroxidation; p.o., oral; I/R, ischemia-reperfusion; HIV, human immunodeficiency virus; RT, reverse transcriptase; IC_50_, inhibitory concentration 50%; SI, selective index=cytotoxic concentration 50%/virus inhibitory concentration 50%; CPE, cytopathic effect; GRO, growth-regulated oncogene; IL, interleukin; ICAM, intercellular adhesion molecule; CK-MB: ISO, isoproterenol; STZ, streptozotocin; Cr, creatinine; BUN, blood urea nitrogen; IFN, interferon; TAC, total antioxidant capacity; NO, nitric oxide; iNOS, inducible nitric oxide synthase; eNOS, endothelial nitric oxide synthase; LPS, lipopolysaccharide; MPO, myeloperoxidase; MMP, matrix metalloproteinase; PG, prostaglandin; COX, cyclooxygenase; STAT, signal transducer and activator of transcription; NF-kB, nuclear factor-κB; WBC, white blood cells; LDH, lactate dehydrogenase; CPK, creatinine phosphokinase; AST, aspartate transaminase; ALT, alanine transaminase; G6PD, glucose 6 phosphate dehydrogenase; LDL, low-density lipoprotein cholesterol; TAG, triacylglycerol; ACE, angiotensin converting enzyme; NPSH, Non protein thiol; RAS, renin angiotensin system; PUFA, poly unsaturated fatty acids; SFA, saturated fatty acid; Ig, immunoglobulin; Casp, caspase; SRBC, sheep red blood cells; DOX, doxorubicin; DNP, dinitrophenyl; HO, heme oxygenase; MCP-1, monocyte chemoattractant protein; VCAM, vascular cell adhesion molecule; RSV, respiratory syncytial virus; BALF, bronchoalveolar lavage fluids; TLR, Toll-like receptor; NQO1, NAD(P)H:quinone oxidoreductase 1; PI3K, phosphatidylinositol-3 kinase; CREB, cAMP response element binding; GSK-3a/b, glycogen synthase kinase 3a/b; ERK, extracellular signal-regulated kinase; As, arsenic; TSH, total sulfhydryl groups; CRP, C-reactive protein; Pb, lead; gGCS, g-glutamylcysteine synthetase; GRP78, glucose−regulated protein 78; PERK, protein kinase RNA−like ER kinase; eIF2a, eukaryotic translation initiation factor−2; JNK, c-Jun N-terminal kinase; AP-1, activator protein-1; CXCL10, interferon-g-inducible protein 10; OVA, ovalbumin; EV, enterovirus; mTOR, mammalian target of rapamycin; RDDP, Reverse Transcriptase-associated DNA Polymerase; RNase H, Ribonuclease H; a-SMA, asmooth muscle actin; STAT, signal transducer and activator of transcription; HSP, heat shock protein; NOX4, NADPH oxidase 4; PPAR, Poly(ADP) ribose polymerase; HMGB, High Mobility Group Box.

*Bold studies are antiviral assessments against viral lung pathogens*.

**Table 3:** Quality assessment of animal studies on the pharmacological activity of traditional Persian medicine-suggested plant possibly beneficial in COVID-19 according to Animal Research: Reporting of *In vivo* Experiments (ARRIVE) guideline.

Reference	Validity	Ethical statement	Animals	Experimental procedures	Housing & husbandry	Numbers analyzed	Interpretation & scientific implications	Generalizability/translation
(Sawamura et al., 2010)	–	+	+	+	+	+	–	–
(Xiong et al., 2012)	–	+	+	+	–	+	–	–
(Hong et al., 2015)	+	+	+	+	–	–	–	–
(Abdel-Daim et al., 2017)	+	+	+	+	+	+	–	+
(Zhao et al., 2019)	+	+	+	+	–	+	–	+
(Yu et al., 2017)		+	+	+	+	+	–	+
(Padiya et al., 2014)	–	+	+	+	+	+	–	–
(Qi et al., 2020)	+	+	+	+	+	+	–	+
(Sachdeva et al., 2012)	+	+	+	+	+	+	–	+
(Mehdizadeh et al., 2013)	+	–	+	+	+	+	–	–
(Razmaraii et al., 2016)	+	+	+	+	+	+	–	+
(Ojha et al., 2013)	+	+	+	+	+	+	–	+
(Li et al., 2019)	+	+	–	+	+	+	–	+
(Thirunavukkarasu et al., 2015)	+	+	+	+	–	–	–	–
(Bhattacharya et al., 2002)	–	–	+	+	+	+	–	+
(Ojha et al., 2012)	+	+	+	+	+	+	–	+
(Patel and Goyal, 2011)	+	+	+	+	+	+	–	+
(Lu et al., 2019)	+	+	+	+	–	+	–	+
(Panda and Kar, 2010)	+	+	+	+	+	+	–	–
(Feki et al., 2019)	+	+	+	+	+	+	–	–
(Mukthamba and Srinivasan, 2015)	+	+	+	+	+	+	–	–
(Tripathi and Chandra, 2009)	+	+	+	+	+	+	–	+
(Pradeep and Srinivasan, 2018)	+	+	+	+	+	+	–	–
(Panda et al., 2013)	+	+	+	+	+	+	–	–
(Ammar el et al., 2013)	+	+	+	+	+	+	–	+
(Cheng et al., 2012)	+	+	+	+	+	+	–	–
(Kim et al., 2002)	+	–	+	+	+	+	–	–
(Lepczynski et al., 2015)	+	+	+	+	–	–	–	–
(Ramadan et al., 2011)	+	+	+	+	–	–	–	+
(Long et al., 2016)	+	+	+	+	+	+	–	–
(Zou et al., 2018)	–	+	+	+	+	–	–	+
(Mohi El-Din et al., 2014)	+	+	+	+	+	–	–	–
(Nie et al., 2019)	+	+	+	+	–	+	–	+
(Mo et al., 2020)	+	+	+	+	+	+	–	+
(Seo et al., 2013)	–	+	–	+	–	+	–	+
(Shu et al., 2014)	–	+	+	+	+	+	–	–
(Zha et al., 2013)	–	+	+	+	+	+	–	+
(Zhang et al., 2020b)	–	+	+	+	+	+	–	+
(Zaghloul et al., 2019)	+	+	+	+	–	+	–	+
(Boskabady et al., 2014)	+	+	+	+	–	+	–	+
(Ma et al., 2013)	+	+	+	+	–	+	–	+
(Nishimoto et al., 2010)	–	+	+	+	–	+	–	+
(Qu et al., 2019)	+	+	+	+	–	+	–	+
(Guan et al., 2016)	+	+	+	+	+	+	–	+
(Song et al., 2019)	–	+	+	+	+	–	–	+
(Shen et al., 2019)	+	+	+	+	+	+	–	+
(Chniguir et al., 2019)	–	+	+	+	–	+	–	–
(Magalhaes et al., 2010)	+	+	+	+	–	+	–	+
(Kandhare et al., 2015)	+	+	+	+	+	+	–	–
(Liu et al., 2017)	+	+	+	+	+	+	–	+
(Hu et al., 2019)	+	–	–	+	–	+	–	+
(Lu et al., 2018)	+	+	+	+	+	+	–	+
(Ahmad et al., 2014)	+	+	+	+	+	+	–	–
(Miltonprabu et al., 2017)	+	+	+	+	+	+	–	+
(Zhu et al., 2017)	+	+	+	+	+	+	–	+
(Sivakumar et al., 2010)	+	+	+	+	+	+	–	–
(Jin et al., 2018)	+	+	+	+	+	+	–	–
(Pourfarjam et al., 2017)	+	+	+	+	+	–	–	–
(Rezaee-Khorasany et al., 2019)	+	+	+	+	+	+	–	–
(Erdemli et al., 2017)	+	+	+	+	+	+	–	+
(Altinoz et al., 2015)	+	+	+	+	+	–	–	–
(Zhao et al., 2016)	+	+	+	+	+	+	–	–
(Malik et al., 2016)	+	+	+	+	+	+	–	+
(Zhang et al., 2018)	+	+	+	+	–	+	–	–
(Tu et al., 2017)	–	+	+	+	+	–	–	+
(Nassan et al., 2015)	–	–	+	+	+	–	–	+
(Pradeep et al., 2019)	+	+	+	+	+	+	–	+
(Gao et al., 2018)	+	+	+	+	–	+	–	+
(Wang C. et al., 2017)	+	+	+	+	+	+	–	+
(Ulusoy et al., 2014)	+	+	+	+	+	–	–	+

## Results

### Amla (*Phyllanthus emblica* L.)

Amla is the fruit of a tree from the family Phyllanthaceae, which is used as fresh fruit, jam, or electuary (Yadav et al., 2017). In TPM, amla fruit is considered as a cardiotonic and cardioprotective medicine that is useful for treating cardiovascular problems. Amla also prevents humors from infection and thus acts as a general tonic, i.e. strengthens the body against infection (Aghili Khorasani, 1771 AD).

The antiviral activity of amla has been demonstrated against the human immunodeficiency virus (HIV) (**Table 2**). Polyphenols such as kaempferol and quercetin glycosides, gallotannins, and putranjivain A, a potent non-competitive inhibitor of HIV reverse transcriptase (RT), are known as the main anti-HIV components of amla. The higher inhibitory activity of putranjivain A (IC_50_ = 3.9 μM) in comparison to the other isolated phytochemicals (IC_50_>200 μM) from this plant seems to be the result of the hexahydroxydiphenoyl functional group (el-Mekkawy et al., 1995).

In addition to the direct antiviral activity of amla which can suggest natural molecular backbones for possible antiviral agents against SARS-COV-2, there are several reports on the protective effects of the plant on the main organs damaged in SARS-COV-2 infection (**Table 2**). Pyrogallol, a small gallotanin of amla, demonstrated *in vitro* protective effect on bronchial epithelial cells of cystic fibrosis infected with *P. aeruginosa*. The compound could significantly prevent bacterial pulmonary inflammation, evident from the reduced production of neutrophil chemokines, pro-inflammatory interleukins (IL), and intercellular adhesion molecule (ICAM)-1, an important contributor in leukocyte chemotaxis (Nicolis et al., 2008).

Additionally, amla could prevent immunotoxicity induced by chromium and arsenic through modulating the phagocytic properties of immune cells, as well as restoring their ability to produce interferon (IFN)-γ, a critical mediator of the immune system (Sai Ram et al., 2002; Sai Ram et al., 2003).

The cardioprotective effect of amla fruit has been demonstrated in several studies (**Table 2**). It has also been reported that it prevents the myocardial depletion of creatine kinase-MB (CK-MB), a marker of cardiac damage, and improves hemodynamic parameters, further showing its cardioprotective effects (Patel and Goyal, 2011; Ojha et al., 2012). Emblicanin-A and B, two small-sized hydrolysable gallotanins, have exhibited a protective effect in ischemia-reperfusion (I/R)-induced cardiac damage in rats. Emblicanins could represent cardioprotective properties mostly through the induction of endogenous enzymatic antioxidant defense mechanisms and the prevention of lipid peroxidation. The effect of 50 mg/kg of this compound was equal to 200 mg/kg of vitamin E (standard antioxidant); whereas 100 mg/kg of emblicanins showed a higher potency compared with vitamin E (Bhattacharya et al., 2002). These compounds have also represented nephroprotective activity in an animal model of cisplatin-induced nephrotoxicity through the same mechanism (Malik et al., 2016). In terms of the cardioprotective effects of amla extract, another target, the phosphoinositide 3-kinase/glycogen synthase kinase 3β (PI3K/GSK3β) pathway, was identified in the I/R model. The phosphorylation of Akt and subsequently GSK3β leads to the release of 1-methyl-4-phenyl-1,2,3,6-tetrahydropyridine and β-catenin nuclear translocation which further activates anti-apoptotic signaling pathways like B cell lymphoma-2 (Bcl-2) and endothelial Nitric oxide synthase (eNOS), finally resulting in cardioprotection (Thirunavukkarasu et al., 2015).

The effective animal dosage of amla is 100-500 mg/kg/day for the aforementioned therapeutic activities. This dose is equal to a relatively high human dose; however, the plant has an acceptable safety profile and is routinely taken in several countries of the world. Thus, these studies suggest that amla could be a functional food, useful for primary and secondary prevention of COVID-19 (Pingali Usharani and Muralidhar, 2013; Upadya et al., 2019; Kapoor et al., 2020). Interestingly, amla fruit is the second richest source of vitamin C (nearly 600-700 mg in each fruit), which WHO has recommended people take to protect the immune system against SARS-CoV-2 infection (Goraya and Bajwa, 2015).

### Chicory (*Cichorium intybus* L.)

Chicory is a cosmopolitan herbaceous plant from the Compositae (Asteraceae) family, used both as a medicinal plant and a food additive because it tastes similar to coffee. All parts of the plant, including roots, aerial parts, and seeds, are used due to its medicinal properties. Chicory is well-known as a hepatoprotective plant in different complementary and alternative medicines (Street et al., 2013). In TPM, it is also considered as a tonic for kidney and heat-related heart diseases, as well as a modulator of overall health through prevention of humor infection.

Chicory extract was effective in the prevention of diabetic nephropathy following three weeks of administration to rats. At the dose of 125 mg/kg, the effect of chicory was approximately equal to 100 mg/kg of metformin in several parameters (Pourfarjam et al., 2017). The nephroprotective effect of the extract was confirmed in the human kidney cell line through inhibition of GLUT-9 expression, an important transporter of uric acid in kidneys. Chicory extract could also decrease renal damage in an experimental model of chronic renal failure; however, the administered doses of the extract (6.6 and 13.2 g/kg) were dramatically higher than benzbromarone (20 mg/kg) as the standard drug (Jin et al., 2018).

Chicory is also demonstrated to have immunomodulatory activities. In the animal model of ethanol-induced immunotoxicity, chicory increased both circulating leukocytes and the weight of lymphatic organs, showing an improvement in immune system function (Kim et al., 2002). Additionally, chicory as a dietary supplement could affect the plasma protein profiles, resulting in the lower level of pro-inflammatory markers such as the C-reactive protein (CRP) (Lepczynski et al., 2015). Chicory root is a rich source of inulin-type fructans, a group of carbohydrates considered as prebiotics (Lepczynski et al., 2015). Today, the remarkable role of the normal flora of different body organs in various diseases, including immunological problems, has attracted the attention of scientists (Pretorius et al., 2018). Thus, aside from the direct immunomodulatory effects of chicory, the presence of such prebiotics in this plant may also have a modulatory effect on normal flora. The indirect protective effect of chicory against pathologic conditions can be hypothesized in future studies.

### Clove (*Syzygium aromaticum* (L.) Merr. & L.M.Perry)

The flower buds of clove, from the family Myrtaceae, have long been used in both medicine and for culinary purposes. The phenylpropanoids of the essential oil, mainly eugenol, are considered to be the main active compounds of the plant and are responsible for several pharmacological activities (Chaieb et al., 2007). Clove is known as a tonic for the cardiovascular system in TPM and is thought to improve blood supply to both the heart and the brain. Moreover, it is mentioned to be specifically useful in chronic coughs, shortness of breath, and palpitations (Aghili Khorasani, 1771).

The methanolic and aqueous extracts of clove have shown inhibitory effects on HCV protease enzyme with an IC_50_ of 33 μg/ml (Hussein et al., 2000). Eugenol has exhibited antiviral effects against the influenza A virus through direct reduction of viral replication, as well as inhibition of autophagy, a supporting mechanism for viral replication and cell death which results in acute lung damage. It should be mentioned that the potency of eugenol at the concentration of 5 μg/ml was equal or higher than 25 μg/ml of ribavirin as the gold standard antiviral agent, recommending eugenol as a potent antiviral compound (Dai et al., 2013). Furthermore, eugenol could regulate cellular inflammatory cascades such as nuclear factor-κB (NF-κB) and extracellular signal-regulated kinase (ERK)/mitogen-activated protein kinase (MAPK) pathways, nitric oxide (NO), the release of pro-inflammatory ILs, and endogenous antioxidant defense mechanisms (**Table 2**).

Clove has demonstrated modulatory effects on the function of murine white blood cells and macrophages damaged through inflammation/oxidative stress damage (**Table 2**). In the animal model of immunosuppression, one-week administration of clove essential oil (400 mg/kg) could improve both humoral and cell-mediated immunity with equal efficacy to 50 mg/kg of levamisole (Carrasco et al., 2009). Clove extract and its major ingredient, eugenol, have shown an anti-inflammatory effect on the lipopolysaccharide (LPS)-induced damage in macrophages. No statistically significant difference was observed between eugenol (100 μg/well) and dexamethasone (10^-4^ mol/l) (Bachiega et al., 2012). The same effect is also reported with a single dose of the flavonoid biflorin (Lee et al., 2016), suggesting this compound is a fast-acting agent that may be useful in acute inflammations such as cytokine storm in COVID-19.

LPS-induced lung inflammation was also relieved with clove aqueous extract and eugenol through reduction of tumor necrosis factor (TNF)-α and inhibition of NF-κB signaling, as well as improvement in alveolar damage (Magalhaes et al., 2010; Chniguir et al., 2019).

Moreover, the aqueous extract has demonstrated a protective effect on an animal model of infectious pyelonephritis (Nassan et al., 2015), a condition also reported in COVID-19 patients (Su et al., 2020).

Aside from the above-mentioned features of clove essential oil, it has shown strong antibacterial effects, even against the infections of immunosuppressed hospitalized patients (Chaieb et al., 2007). Thus, the essential oil can be a valuable option to prevent secondary bacterial infections in COVID-19 patients.

### Damask Rose (*Rosa × damascena* Herrm.)

Damask rose, from the family Rosaceae, is one of the most valued medicinal plants in TPM due to its modulatory effects on the function of almost all body organs and consequently, overall health (Nayebi et al., 2017). It helps the body to excrete abnormal watery phlegm humor which is highly susceptible to infection and thus, it is a tonic of the lungs. Furthermore, TPM texts discuss that it alleviates infectious fevers (Aghili Khorasani, 1771).

An *in vitro* study on the antiviral properties of damask rose has demonstrated significant activity against HIV infection. Flavonoids purified from the methanolic extract including quercetin, kaempferol, and two of its analogues, showed the highest activity *via* the inhibition of viral protease and gp 120 binding to CD4 glycoprotein. The compounds were not as potent as azidothymidine (zidovudine) as a standard anti-HIV agent in regard to SI and IC_50_; however, they showed an additive effect in combination with this drug (Mahmood et al., 1996).

Damask rose has shown cardiotonic properties on perfused guinea pig heart and reversed bradycardia by increasing heart contractility (Boskabady et al., 2013). Both ethanolic extract and the essential oil represented an antispasmodic effect on guinea pig tracheal chains (Boskabady et al., 2006).

### Fenugreek (*Trigonella foenum-graecum* L.)

Fenugreek is a member of the family Leguminosae and the seeds are frequently used as a lung tonic in TPM due to the moderate heat, causing a mucolytic activity on pulmonary mucosa. This effect helps to remove the thick phlegm humor and causes a soothing effect on lung injuries, accelerating the healing procedure (Aghili Khorasani, 1771).

Fenugreek seed extract has demonstrated significant anti-inflammatory properties in an animal model of pulmonary inflammation, evident from the reduction of leukocytes infiltration to the bronchoalveolar lavage fluid (BALF) and lung fibrosis. Pro-inflammatory cytokines and endogenous enzymatic and non-enzymatic antioxidants were also restored to near normal levels. Stimulation of nuclear factor E2-related factor 2 (Nrf2) by fenugreek, an antioxidant cascade, prevents the hemeoxigenase-1 (HO-1) overproduction, subsequently ameliorating pulmonary fibrosis. The effectiveness of the extract with 5-40 mg/kg dose range in most of the investigated parameters were equal or higher than 10 mg/kg of methylprednisolone as the gold standard drug (Kandhare et al., 2015). Likewise, the antiasthmatic effect of fenugreek seed syrup, designed based on the lung protecting properties of the plant in TPM, was assessed in a clinical trial. Four weeks of treatment with the syrup caused a significant improvement in spirometry parameters in comparison to the baseline values (Emtiazy et al., 2018).

There are also several studies on the cardioprotective effects of fenugreek (**Table 2**). Dietary fenugreek powder (Mukthamba and Srinivasan, 2015), trigonelline (a pyridine alkaloid), and digoxigenin-3-O-rutin (a cardiac glycoside) have reversed isoproterenol-induced cardiotoxicity in rats (Panda and Kar, 2010; Panda et al., 2013). Both compounds exerted modulatory effects on the cardiac biomarkers of oxidative stress and inflammation, including CK-MB, lactate dehydrogenase (LDH), and lipid peroxidation. Trigonelline showed the highest pharmacological activity at a dose of 40 mg/kg. On the other hand, digoxigenin-3-O-rutin was effective at 2.5-10 mg/kg which was comparable with 5 mg/kg of digoxin as the gold standard drug (Panda and Kar, 2010). Furthermore, trigonelline could reduce the level of heat shock protein (HSP)-27 and αB-crystallin, two novel biomarkers of oxidative damage, in the myocardium damage, further confirming the cardioprotective activity of fenugreek (Panda et al., 2013). Polysaccharides as another important category of fenugreek components have demonstrated significant cardioprotective activity both *in vitro* and *in vivo*, possibly due to their antioxidant activity and prevention of DNA damage (Feki et al., 2019). Aside from antioxidant activity (Tripathi and Chandra, 2009), inhibition of pathologic NO production by inducible nitric oxide synthase (iNOS) and markers of cardiac fibrosis, such as fibronectin and collagen, seem to be the main cardioprotective mechanisms of fenugreek demonstrated in streptozotocin (STZ)-induced cardiac damage (Pradeep and Srinivasan, 2018).

In a cyclophosphamide-induced animal model of immunosuppression, fenugreek could prevent lymphopenia and neutropenia, and improve the cellularity of bone marrow, spleen, and thymus, proposing an immunostimulatory effect for this plant (Ramadan et al., 2011).

### Galangal (*Alpinia galanga* (L.) Willd., *A. officinarum* Hance)

Galangal species belong to the family Zingiberaceae, a valuable plant family comprising of several important medicinal plants such as ginger and turmeric (Abubakar et al., 2018). In TPM, the plant is known to have nephroprotective properties and lung tonic activity and is used for the treatment of cough. Aside from the essential oil, the most important secondary metabolites in this plant family are diarylheptanoids with significant anti-inflammatory properties (Abubakar et al., 2018).

Seven diarylheptanoids from lesser galangal (*A. officinarum*) have demonstrated significant *in vitro* antiviral effects on RSV, poliovirus, and measles virus. The lowest IC_50_ values were 5, 3.7, and 6.3 μg/ml against RSV, poliovirus, and measles virus, respectively; however, they were all higher than those of the gold standards, ribavirin and acyclovir (Konno et al., 2011). In another study, the antiviral effects of two galangal diarylheptanoids were assessed against several types of influenza virus, one of which showed remarkable activity. The active compound was not only effective *in vitro* against all virus types, including oseltamivir-resistant type, but also showed *in vivo* protective effects on the murine model of influenza. The compound showed a dose-dependent inhibition of viral RNA and antigen expression; while it was ineffective on the viral adsorption or invasion. The *in vitro* inhibition of viral growth by 60 μg/ml of the compound was higher than 20 μg/ml of ribavirin (Sawamura et al., 2010). 1ʹ-Acetoxychavicol acetate, a phenylpropanoid from the greater galangal (*A. galanga*) also exhibited anti-influenza activity with an IC_50_ of 2 μM (460 ng/ml). This effect was mediated through the inhibition of viral ribonucleoprotein complex nuclear export, an important part of the viral life cycle that controls the transcription and replication (Watanabe et al., 2011). Furthermore, the compound has demonstrated an inhibitory effect on the nuclear export of HIV-Rev protein. Analysis of the structure-activity relationship revealed that the presence of 10-acetoxyl-20-ene moiety, two acetyl functional groups, along with a 10-S configuration is crucial for its antiviral activity (Tamura et al., 2009). It is worth mentioning that a molecular docking analysis showed the effectiveness of galangal compounds against SARS-CoV-2; however, experimental studies are needed to confirm this hypothesis (Zhang et al., 2020a).

1ʹ-Acetoxychavicol acetate has been proved to have a protective effect on the lungs, as well. In an ovalbumin-induced mouse model of asthma, the compound reduced the infiltration of eosinophils into the BALF. The secretion of pro-inflammatory cytokines by both types 1 and 2 T cells was also significantly decreased and the effect of the higher dose (50 mg/kg) was equal to 1 mg/kg of dexamethasone (Seo et al., 2013). Galangin, a flavonoid of the greater galangal, has represented anti-inflammatory effects in the same animal model *via* inhibition of the NF- κB pathway. Furthermore, galangin decreased the monocyte chemoattractant protein (MCP)-1 and vascular cell adhesion molecule (VCAM)-1 of lung tissue, both of which participate in leukocytes chemotaxis (Zha et al., 2013). It should be mentioned that in most evaluated parameters, the effect of 15 mg/kg of galangin was equal or higher than dexamethasone (3 mg/kg), showing a high anti-inflammatory potency (Zha et al., 2013). Moreover, galangin has demonstrated anti-inflammatory effects in LPS-induced acute lung damage (Shu et al., 2014) and macrophage stimulation (Jung et al., 2014) through the suppression of NF-κB downstream signaling.

In addition to galangin, 1ʹ-acetoxychavicol acetate has also shown immunoregulatory properties in stimulated murine macrophages *via* the prevention of NF-κB activation and IFN-β mRNA expression, subsequently inhibiting NO production by iNOS. Despite the important role of NO in physiological status, its overproduction by iNOS is involved in several pathologic inflammatory conditions (Ando et al., 2005).

Likewise, cardamonin, another flavonoid from *A. galanga*, decreased the cardiotoxicity of doxorubicin (DOX) *via* inhibition of both inflammation and oxidative stress through the Nrf2 pathway. Nrf2 has a close cross-talk with NF-κB and thus, the antioxidant and anti-inflammatory effect of galangal *via* these pathways is further confirmed (Qi et al., 2020).

Galangin exhibited nephroprotective effects in the high-fructose diet-induced renal damage by inhibition of oxidative damage (Sivakumar et al., 2010).

Taken together, galangal species seem to have direct antiviral properties, as well as protective effects on the main organs damaged in SARS-CoV-2 infection and may be suitable complementary therapies in this infection.

### Garlic (*Allium sativum* L.)

Even though garlic, from the family Amaryllidaceae, is one of the most ancient medicinal plants there is always has something new to say about it medicinally. In TPM, it is useful for the primary and secondary prevention of different infections and is recommended that it be used during epidemic infectious diseases. Moreover, it has been proposed as a blood thinner and used for the management of several types of cardiovascular events (Aghili Khorasani, 1771). Garlic owes several of its significant pharmacological activities to the organosulfur compounds which are also responsible for the strong flavor and fragrance of the plant (Li et al., 2013).

A recently published molecular docking analysis demonstrated the high inhibitory effects of garlic volatile organosulfur compounds on the invasion of SARS-CoV-2. This effect was mediated through the inhibition of ACE2, a participant in SARS-CoV-2 infection. Allyl disulfide and allyl trisulfide, the major components of garlic essential oil, showed the highest antiviral activity (Thuy et al., 2020). Garlic has demonstrated therapeutic activity against the Dengue virus, a member of the Flaviviridae family causing a lethal hemorrhagic fever. Diallyl disulfide, diallyl sulfide, and alliin could decrease the inflammatory markers in infected cells through the inhibition of oxidative damage (Hall et al., 2017). There are several other reports on the antiviral effects of garlic against influenza viruses A and B, rhinovirus, rotavirus, HIV, and viral pneumonia. Most of these investigations date back to more than twenty years ago when current, more precise techniques were not available (Bayan et al., 2014). Thus, the results of these antiviral assessments need to be reconfirmed with newly developed laboratory methods.

Garlic aqueous extract could effectively ameliorate pulmonary interstitial alveolitis and macroabscesses in lung damage (Mohi El-Din et al., 2014). In LPS-induced acute pulmonary inflammation, oral administration of S-allylmercaptocysteine could dose-dependently inhibit lung damage. The effect of 60 mg/kg of S-allylmercaptocysteine was equal to or higher than 500 mg/kg of N-acetylcysteine (positive control), showing a high potency. This compound could suppress pro-inflammatory cytokines, reducing macrophage and neutrophils infiltration into BALF, inhibiting NF-κB activation, and improving endogenous enzymatic and non-enzymatic antioxidants. Furthermore, Nrf2 and its downstream signals, HO-1 and NAD(P)H: quinone oxidoreductase 1 (NQO1), a cytoprotective mediator in oxidative damage, were increased by S-allylmercaptocysteine (Mo et al., 2020). The effect of garlic essential oil and organosulfur components on human neutrophils was also demonstrated *in vitro*, where they could improve neutrophils function as an immunomodulatory response (Schepetkin et al., 2019). Moreover, S-allyl-L-cysteine has shown inhibitory effects on bleomycin-induced pulmonary inflammation and fibrosis at 10 mg/kg; though, the potency cannot be accurately judged, since no positive control drug was used (Nie et al., 2019). In addition to animal studies, aged garlic extract (garlic soaked in alcohol) has represented beneficial properties in reducing the number and duration of symptoms in subjects with cold/flu. These results also showed an improved proliferation of natural killer (NK) cells and γδ-T lymphocytes in response to pathogen-associated molecular patterns (Nantz et al., 2012).

In another study, black garlic, another popular product prepared through garlic fermentation, was compared with fresh raw garlic regarding its immunostimulatory effects on macrophages. While fresh garlic could significantly improve the phagocytic activity of macrophages and production of cytokines, black garlic showed only negligible effects on these parameters. This significant variation is attributed to the different polysaccharides in the two extracts, specifically fructans which are degraded in black garlic (Li et al., 2017).

Garlic is also widely used for different cardiologic problems and myocardial protection (Bradley et al., 2016). In this regard, raw garlic homogenate showed protective effects on fructose-induced oxidative stress in cardiac tissue *via* the elevation of cardiac H_2_S and preventing myocardial injury. Additionally, PI3K/AKT signaling, a critical pathway in cell survival linked with Nrf2, was activated by garlic (Padiya et al., 2014). Alliin has demonstrated modulatory effects on autophagy, a mechanism involved in cytoprotection and apoptosis of different cells, including myocardium (Zhao et al., 2019). Similarly, allicin has exhibited anti-inflammatory and antioxidant properties in DOX-induced cardiotoxicity (Abdel-Daim et al., 2017). Allicin is produced from alliin by alliinase after chopping garlic and is further metabolized into diallyl trisulfide. The latter compound has exerted cardioprotective effects through AMP-activated protein kinase (AMPK), GSK-3β, and hypoxia-inducible factors (HIF)-1α (Yu et al., 2017). Considering the doses of alliin, allicin, diallyl trisulfide (20, 100 mg/kg) versus garlic homogenate (250 mg/kg), it can be hypothesized that the purified compounds are more potent than garlic. However, a study on all of these components in the same setting is required to clarify this hypothesis.

Diallyl trisulfide has also exhibited nephroprotective activity in arsenic-induced kidney damage. In addition to the improvement of BUN and creatinine as routine markers of renal function, the compound could modify several markers of inflammation, apoptosis, and oxidative stress (**Table 2**) (Miltonprabu et al., 2017). A similar activity was observed with *in vitro* and *in vivo* administration of S-allylmercaptocysteine in cisplatin-induced kidney injury. Furthermore, p-53, an initiator of apoptosis, and its downstream proteins B-cell associated X protein (Bax) and Bcl-2 were regulated by this compound. Poly ADP ribose polymerase (PARP), a DNA repairing enzyme during apoptosis which is also under regulation of p-53, was decreased as well, revealing the reversal of cisplatin-induced DNA damage (Zhu et al., 2017).

In general, garlic and its organosulfur compounds act as a multitargeted therapy in several tissues susceptible to SARS-CoV-2 injury and may be beneficial as a primary/secondary prevention in these patients.

### Grape and Raisin (*Vitis vinifera* L.)

Grape, from the family Vitaceae, is one of the most respected fruits in TPM since it produces a series of physiologically balanced humors and thus, is recommended for the general population. In addition to its high nutritional value, modern pharmacological investigations have focused on the remarkable antioxidant properties of this fruit. This attention is due to polyphenolic compounds including proanthocyanidins in the flesh and resveratrol in the fruit seed/peel (Nassiri-Asl and Hosseinzadeh, 2016). Intriguingly, it is emphasized in TPM to take grape with its seed for the urologic problems, showing the knowledge of Persian physicians about the specific effects of the seeds.

In a virtual screening considering influenza A virus vRNA promoter, as well as an *in vitro* evaluation, procyanidin, a major component of grape extract, revealed significant antiviral activity (Dai et al., 2012). Grape seed extract has shown inhibitory effects on HCV replication *via* suppression of virus-induced cyclooxygenase (COX)-2 overexpression. It has also shown a synergistic effect by co-administration with conventional anti-HCV medicines including telaprevir, daclatasvir, sofosbuvir (Chen et al., 2016). Suppression of the MAPK/JNK pathway is also involved in the antiviral effect of grape seed extract against both HCV (Chen et al., 2016) and RSV (Lee et al., 2017). Moreover, the extract has prevented ILs, MAPK/JNK, and NF-κB pro-inflammatory cascades and regulated mucin production *via* reducing the expression of several mucin MUC genes in the airway epithelium. Interestingly, this effect is in line with the lung protecting properties of grape as described in TPM, by cleaning the pathological viscous pulmonary mucosa (Lee et al., 2017; Kim et al., 2019).

In lead and arsenic-induced lung inflammation, the extract reduced pulmonary levels of pro-inflammatory cytokines, oxidative damage, and pro-apoptotic markers (Lu et al., 2018; Hu et al., 2019). In carrageenan-induced acute lung inflammation, a single oral dose of grape seed extract could significantly balance the level of several pro-inflammatory and anti-inflammatory cytokines. In addition, it modulated tumor necrosis factor receptor, a co-activator of effector T lymphocytes, upregulated during lung inflammation (Ahmad et al., 2014). In pulmonary fibrosis induced by bleomycin, the extract prevented leukocytes infiltration and markers of fibrosis including matrix metalloproteinase and collagen deposition and increased the anti-fibrotic marker E-cadherin. The potency of the higher extract dose, 100 mg/kg, was equal to 0.5 mg/kg of dexamethasone in most parameters (Liu et al., 2017).

The immunomodulatory effect of grape was assessed in the subchronic immunotoxicity by aflatoxin B1, a fungal toxin causing oxidative stress and subsequent cellular damages. Grape seed extract at the dose of 100 mg/kg showed a significant improvement of endogenous antioxidants and decreased pro-inflammatory cytokines to a similar level to those of the healthy animals (Long et al., 2016).

The cardioprotective effect of the extract was also demonstrated in DOX-induced cardiotoxicity, evident from the modulation of electrocardiogram (ECG) and an improvement of endogenous antioxidants activity (Ammar el et al., 2013). Another specific cardioprotective mechanism that has been reported for grape proanthocyanidins is the modification of protein kinase RNA−like ER kinase (PERK) and eukaryotic translation initiation factor−2 (eIF2α). In endoplasmic reticulum oxidative stress conditions, overactivation of PERK/eIF2α stimulates C/EBP−homologous protein (CHOP) and subsequently, apoptosis. Grape seed extract could reverse the I/R-induced upregulation of these pathways and prevent the apoptosis of cardiomyocytes (Wang X. et al., 2017).

The inhibition of endoplasmic reticulum-dependent apoptosis was also involved in the nephroprotective effects of grape in diabetic nephropathy (Gao et al., 2018). In another study in diabetic nephropathy, procyanidin B2 could exhibit antioxidant activity *via* the prevention of mitochondrial dysfunction, a phenomenon accompanied by increased production of ROS as the byproduct of cell metabolism (Bao et al., 2015). Grape seed extract has represented protective effects against kidney damage *via* the reduction of caspase enzymes, an important category of participants in apoptosis, as well as inhibition of endothelial and inducible forms of NOS. The potency of 100 mg/kg of the grape extract was equal or higher than the same dose of N-acetylcysteine as the positive control (Ulusoy et al., 2014). The extract has also shown anti-inflammatory properties against arsenic-induced nephrotoxicity through the regulation of pro-inflammatory and anti-inflammatory cytokines, as well as deactivation of NF-κB (Wang C. et al., 2017).

Considering the above-mentioned beneficial effects of grape, in addition to its nutritional value and safety, we suggest that this fruit to be part of the diet of patients with COVID-19. Since the fresh fruit, having several minerals and natural vitamins, is easily available in almost all parts of the world, the juice can be taken orally or even administered *via* nasogastric tube.

### Jujube (*Ziziphus jujuba* Mill.)

Jujube fruit from the family Rhamnaceae is one of the valuable, yet not well-recognized medicinal foods mostly grown in Iran and China (Shahrajabian et al., 2019). It is a popular plant as a traditional remedy for different types of coughs. In TPM, it is mostly considered as a modulator of the quality of humor, mainly by preventing the negative effect of excess heat. It is also described as having soothing effects on pulmonary inflammations and shortness of breath (Aghili Khorasani, 1771). In addition to its high nutritive value, jujube is a rich source of bioactive secondary metabolites such as polyphenols, polysaccharides, and terpenoids (Gao et al., 2013).

Betulinic acid, a triterpene constituent of jujube fruit, has shown antiviral activity against influenza A virus both *in vitro* and *in vivo*. At the concentration of 50 μM, betulinic acid showed a 98% inhibition of virus cytopathic effects; while it was not toxic for the host cells. Despite the remission of symptoms in the infected animals, no significant change was observed in viral replication and pro-inflammatory cytokines (except for IFNγ). Thus, further mechanistic investigations are needed to establish the antiviral properties of this compound (Hong et al., 2015).

Acidic polysaccharides are another category of active phytochemicals of jujube fruit with immunostimulatory properties. These effects were evident from the increased indices of main lymphatic organs, i.e. spleen and thymus in animals, showing an improved proliferation of immune cells. Additionally, these polysaccharides contain a series of metal ions which possibly participate in biological activities (Zou et al., 2018).

Two categories of polyphenols of jujube peel, free phenols, and bond phenols, were assessed regarding their cardioprotective activity in ISO-induced heart injury in rats. Both types of phenols demonstrated prophylactic antioxidant properties and modulatory effects on different cardiac ion channels with no significant difference between the two polyphenol categories (Cheng et al., 2012). Jujuboside A, another major triterpenoid of this plant, has represented *in vitro* cardioprotection *via* regulating of the PI3K/AKT/mTOR autophagic pathway (Han et al., 2016).

Despite the valuable properties of the jujube plant, the number of high-quality studies on the different medicinal aspects of this plant are limited. Further investigations regarding the active components of the plant and their mechanisms of action are necessary.

### Licorice (*Glycyrrhiza glabra* L., *G. uralensis* Fisch.)

Licorice from the family Leguminosae is a valuable medicinal plant in TPM, as well as several other doctrines of traditional medicines. The sweet taste of the roots is mostly due to the triterpenoid glycyrrhizin and the plant has several valuable secondary metabolites such as saponins and flavonoids (Pastorino et al., 2018). In TPM, licorice is highly respected as an antitussive medicine for different types of coughs and lung diseases and is also recommended for chronic fevers due to infections (Aghili Khorasani, 1771).

Licorice is a well-studied plant in terms of its antiviral activity (Wang et al., 2015). In addition to its remarkable antiviral properties against some RNA-viruses like HCV and HIV (Adianti et al., 2014; Fukuchi et al., 2016), licorice has demonstrated remarkable antiviral effects on respiratory viruses. Glycyrrhizin has shown a high inhibitory effect on the *in vitro* replication of two clinical isolates of SARS (Cinatl et al., 2003). Accordingly, fifteen semisynthetic derivatives of this compound were virtually screened against the virus. Glycoside, amide, and carboxyl moieties can significantly increase the activity in comparison to the original backbone; however, the cytotoxicity was also increased in these new structures (Hoever et al., 2005). In influenza A H5N1-infected lung cells, glycyrrhizin inhibited both viral replication and host cell inflammatory and apoptotic response to the infection, a condition responsible for severe flu symptoms (Michaelis et al., 2011). In silico analysis has demonstrated the ability of twelve licorice components to inhibit influenza neuraminidase (NA), a viral surface enzyme involved in the release of replicated viruses from infected host cells (Grienke et al., 2014). Aqueous licorice extract, glycyrrhizin, and one of its metabolites, 18β-glycyrrhetinic acid were investigated against human RSV. While the extract and 18β-glycyrrhetinic acid revealed significant antiviral activity, glycyrrhizin was inactive (Feng Yeh et al., 2013). This observation forms the hypothesis that glycyrrhizin acts as a pro-drug which turns into active metabolites such as 18β-glycyrrhetinic acid but future investigations are required to examine this idea.

Licorice has been traditionally used for cough in the form of a medicinal candy or lozenge. In a clinical study, patients who underwent thoracic surgery and post-operative double-lumen endotracheal intubation gargled a liquid licorice preparation as a prophylaxis for post-extubation coughing. The incidence of cough and sore throat was significantly lower with licorice compared with placebo (simple sugar syrup) (Ruetzler et al., 2013). In ovalbumin-induced lung inflammation, glycyrrhizic acid caused a dose-dependent regulation of cytokine production by types 1 and 2 of helper T lymphocytes, and the effect of the highest dose (40 mg/kg) was equal to 2 mg/kg of dexamethasone in several parameters (Ma et al., 2013). In LPS-induced inflammation in murine macrophages, glycyrrhizic acid increased autophagy markers such as LC3-II/I and Beclin-1 *via* PI3K/AKT/mTOR pathway. Likewise, the compound demonstrated *in vivo* protective effects on LPS-induced pulmonary inflammation through the reduction of pro-inflammatory cytokines. It also decreased the high mobility group box (HMGB)-1, a product of damaged cells further activating pro-inflammatory pathways (Qu et al., 2019). The same mechanism was also reported for glycyrrhizin (Kim et al., 2015). Moreover, glycyrrhizin represented a stimulatory effect on the nuclear translocation of Nrf2 and HO-1 expression in macrophages *via* the p38 MAPK pathway, an important intracellular signaling modulating apoptosis and autophagy in response to pathologic conditions (Kim et al., 2015). Glycyrrhizin could reduce abnormally-increased mucus production both *in vitro* and *in vivo via* suppression of MUC5AC mRNA expression, confirming the mucolytic properties of licorice mentioned in TPM. The effect of 45 mg/kg of glycyrrhizin on goblet cell hyperplasia was nearly equal to 1 mg/kg of dexamethasone, while 135 mg/kg of this compound was higher than dexamethasone (Nishimoto et al., 2010).

*G. glabra* extracts have shown cardioprotective properties both *in vitro* and *in vivo via* the improvement of endogenous antioxidant defense mechanisms (Ojha et al., 2013; Upadhyay et al., 2020). In DOX-induced toxicity in cardiomyocytes, licorice aqueous extract has improved sirtuin (SIRT)-1, a cardioprotective transcription factor, and its downstream proteins, peroxisomes proliferator-activated receptors (PPAR)-α/γ (Upadhyay et al., 2020). Glycyrrhizic acid has demonstrated an inhibitory effect on the long-lasting (L)-type calcium channels of cardiomyocytes. Although this compound could prevent calcium overload in ISO-induced cardiotoxicity, its potency was lower than verapamil even at the highest (20 mg/kg) dose (Li et al., 2019). Both hydroethanolic licorice extract and glycyrrhizic acid decreased the release of CK-MB and LDH, two indicators of myocardial damage (Ojha et al., 2013; Li et al., 2019).

Glycyrrhizic acid has also exhibited a nephroprotective effect on the LPS-induced renal inflammation *via* modulation of several apoptosis markers such as Bax, Bcl-2, and caspase-3, as well as the pro-inflammatory cytokines and iNOS activity. The reduction of COX-2 activity and its product, prostaglandin E2 (PGE2), was also involved in the nephroprotective activity of this compound (Zhao et al., 2016).

From the above-mentioned studies, it can be inferred that licorice is a multipotential medicinal plant with both direct antiviral properties and protective effects on vulnerable organs in SARS-CoV-2 infection and thus, can be further investigated as an adjuvant therapy in this disease.

### Rhubarb (*Rheum palmatum* L., *R. officinale* Baill.)

Several species of the genus *Rheum* from the family Polygonaceae are commonly consumed as rhubarb. More than the purgative effects that make it a suitable detoxifying agent, rhubarb has pulmonary tonifying activity from the view of TPM. It helps the body to excrete abnormal (pathologic) phlegm and consequently, reduces the organ’s susceptibility to infections (Aghili Khorasani, 1771; Zheng et al., 2013).

*R. palmatum* extract has demonstrated antiviral activity against CVB3, the main reason for viral myocarditis. Interestingly, the viral titers in the internal organs of infected animals treated with 0.3 g/kg of the extract were lower than those that received 0.01 g/kg ribavirin as the gold standard. Serum viral RNA was not detected on the last day of the experiment in the extract-treated group; while it remained positive in the ribavirin group (Xiong et al., 2012). Rheum emodin, an anthraquinone of rhubarb, has also shown antiviral activity against EV-71, mostly through the suppression of viral maturation and virulence and to a lower extent, viral genome levels and protein expression (Zhong et al., 2017). Sennosides A and B, two other anthraquinones of rhubarb, as well as the extracts of two rhubarb species, *R. palmatum* and *R. officinale*, were assessed against HIV infection. The compounds were assessed regarding their inhibitory effects on three main HIV-1 enzymes, i.e. reverse transcriptase (RT)-associated DNA polymerase (RDDP), integrase, and ribonuclease H (RNase H). *R. officinale* extract and sennoside A showed a higher potency toward the inhibition of viral RNase H. Additionally, sennoside A had a lower IC_50_ for RDDP and integrase, showing a higher activity against viral replication which was also confirmed in the cell-based assay (Esposito et al., 2016).

Rhein, another anthraquinone structure of rhubarb, has shown protective effects on RSV-induced lung infection *via* modulation of the host inflammatory response, evident from the reduced levels of pro-inflammatory cytokines and NLRP3. NLRP3 inflammasome is a downstream protein complex of the NF-κB pathway, activating caspase-1 and consequently, apoptosis. It should be mentioned that rhein at a dose of 120 mg/kg showed similar efficacy to 46 mg/kg of ribavirin (Shen et al., 2019). In both *in vitro* and *in vivo* models of pulmonary fibrosis, emodin caused a decrease in α-smooth muscle actin and collagen production. Furthermore, the compound reduced TGF-β1-dependent phosphorylated Smad2/3, a pro-fibrotic mediator inhibiting fibroblast differentiation to myofibroblasts (Guan et al., 2016). Signal transducer and activator of transcription (STAT)-3, another TGF-β1-dependent stimulator of fibroblast activation, as well as HSP-47, a collagen-specific heat shock protein and an indicator of pulmonary fibrosis, were also decreased by emodin, further showing its antifibrotic activity (Guan et al., 2016). Chrysophanol, another anthraquinone from rhubarb, has proved anti-inflammatory properties mostly *via* the inhibition of NF-κB activity and the prevention of lung fibrosis. Moreover, this compound could reduce ovalbumin-induced autophagy and inflammation (Song et al., 2019). In a randomized, controlled clinical trial in patients with acute respiratory distress syndrome (ARDS), a life-threatening condition also observed in COVID-19, a liquid rhubarb preparation was administered *via* nasogastric tube for one week. In comparison to the control group, the adjuvant rhubarb administration could significantly improve oxygenation. Additionally, it could decrease the extravascular lung water index and pulmonary vascular permeability index (He et al., 2017), confirming the results of the preclinical studies in a clinical setting. It should be noted that no identification process was considered for rhubarb and thus, it is not clear which species of the plant were used in this trial (He et al., 2017).

Rhubarb anthraquinones have also demonstrated cardioprotective properties. Emodin could increase the secretion of atrial natriuretic peptide (ANP), a molecule secreted by cardiomyocytes with a multitargeted role in cardioprotection (Zhou et al., 2014). Rhein could restore the downregulation of the PI3K/GSK3β cardioprotective pathway in I/R-induced cardiotoxicity (Liu et al., 2018). Chrysophanol has shown an inhibitory effect on PARylation, the process of PARP attachment to its target proteins, overactivation of which participates in DOX-induced cardiotoxicity. In addition, apoptotic markers, as well as mitochondrial damage were reduced by chrysophanol both *in vitro* and *in vivo* (Lu et al., 2019).

Rhubarb extract could inhibit adenine-induced renal damage *via* the suppression of the TGF-β/Smad pathway (Zhang et al., 2018), a mechanism also reported for its protective effects on the lung (Guan et al., 2016). Furthermore, several other fibrosis biomarkers including E-cadherin, collagen, α-smooth muscle actin and vimentin were decreased in rats treated with rhubarb (Zhang et al., 2018). The aqueous *R. palmatum* extract and rhein have demonstrated nephroprotective activity in cellular and animal models of chronic kidney disease. This effect was mediated *via* suppression of autophagy-related pathways and renal fibrosis (Tu et al., 2017).

The result of preclinical studies on rhubarb show the pharmacological activity of this plant to be highly attributed to its anthraquinones through several therapeutic targets in organs damaged in SARS-CoV-2 infection. Especially, a clinical study of the protective effects of this plant in patients with ARDS represents the significant efficacy and acceptable safety of its use in treating a condition remarkably similar to COVID-19. Thus, rhubarb could be one of the possible choices that are clinically assessed in treating this disease.

### Saffron (*Crocus sativus* L.)

Saffron, known as the red gold, is the stigma of an herbaceous plant from the family Iridaceae, native to Iran, with several pharmacological activities. The most investigated components of the plant are carotenoid structures including crocins and their metabolites, crocetin responsible for the saffron color. The terpene glycoside picrocrocin, as well as the monoterpene aldehyde safranal, a volatile compound causing the specific saffron aroma are other important components of saffron (Boskabady and Farkhondeh, 2016). In TPM, Saffron is considered to be a tonic of the lung and kidneys. It is highly valued not only as a cardiotonic medicine, but also as a means of enhancing the delivery of other medicinal ingredients to the heart. Thus, saffron is one of the most frequently used ingredients in TPM multicomponent preparations for heart diseases (Aghili Khorasani, 1771; Sadati et al., 2016).

The aqueous extract of saffron, as well as crocin and picrocrocin, were evaluated in terms of *in vitro* antiviral activity against HIV-1. Both carotenoids showed antiviral activity with a relatively low IC_50_ (5 and 8 μM) and high SI (>187 and 600)(**Table 2**), showing these compounds to be potent antiviral agents. On the other hand, the aqueous extract showed no considerable antiviral activity, revealing that the antiviral activity of the plant is mostly due to its lipophilic compounds such as carotenoids (Soleymani et al., 2018).

Safranal has demonstrated anti-inflammatory effects on OVA-induced airway inflammation *via* modulation of type 1 and 2 helper T lymphocytes balance, evident from the reduced serum IL-4 and elevated IFNγ (Boskabady et al., 2014). This modulatory effect on cytokine production was also observed *in vitro* in T lymphocytes (Boskabady et al., 2011). Interestingly, even the effect of the lowest dose (4 μg/ml of drinking water) was higher than 50 μg/ml of dexamethasone, showing a significantly higher potency for this compound in alleviating pulmonary inflammation (Boskabady et al., 2014). In bleomycin-induced pulmonary damage, crocin has reduced tissue inflammation *via* the reduction of pro-inflammatory cytokines and markers of fibrosis, improvement of endogenous antioxidant mechanisms, and the induction of the Nrf2 cytoprotective pathway. Likewise, the compound was an inhibitor of Toll-like receptor (TLR)-4, a receptor participating in leukocytes infiltration, neutrophils activation, TNF-α-dependent inflammation, and TGF-β-dependent fibrosis. The antifibrotic effect of crocin at 20 mg/kg was higher than 0.2 mg/kg of halofuginone (Zaghloul et al., 2019). Additionally, crocin has been shown to relieve the effects of LPS-induced acute lung injury by suppressing NF-κB and MAPK pro-inflammatory cascades. Matrix metalloproteinase 9 (MMP-9), heparanse, and two glycocalyx shedding enzymes overactivated in inflammatory lung diseases were also decreased by crocin (Zhang et al., 2020b). In a randomized, triple-blind, placebo-controlled clinical trial in patients with mild to moderate asthma, a saffron powder supplement was administered for two months. Spirometry parameters, including forced vital capacity (FVC), forced expiratory volume in the first second (FEV1), FEV1/FVC ratio, and forced expiratory flow 25-75% were significantly improved in comparison to placebo. Furthermore, serum levels of CRP and the anti-HSP70 antibody were significantly decreased. A direct correlation between the severity of asthma symptoms and anti-HSP70 antibody was observed and thus, its reduction is an indicator of decreased pulmonary inflammation (Hosseini et al., 2018).

Saffron aqueous extract exhibited cardioprotective effects on an animal model of ISO-induced cardiac damage *via* reduction of CK-MB and LDH leakage from myocardial cells and improvement of endogenous antioxidants in cardiac tissue (Sachdeva et al., 2012). A similar result was obtained with safranal from a significantly lower effective dose in comparison to the aqueous extract, suggesting these effects are partially due to this saffron component (Mehdizadeh et al., 2013). In DOX plus I/R-induced cardiotoxicity, saffron could restore the level of contractile proteins including α-actinine, myosin light chain, and troponine C. It could prevent mitochondrial dysfunction and recover the phosphorylation level of the AKT/P70S6K and ERK1/2 cardioprotective pathways (Chahine et al., 2016). These effects may be partially mediated by crocin since this compound has shown to have protective activity with respect to ECG in an animal model of DOX-induced cardiotoxicity (Razmaraii et al., 2016). Similarly, crocin was effective in the reduction of LPS-induced cardiotoxicity *via* a decrease in the protein level and gene expression of pro-inflammatory cytokines. COX-2 enzyme and its product, PGE2, which are increased in LPS-induced damages were returned to the normal level by crocin administration (Rahim et al., 2019).

Saffron extract has shown detoxifying effects on alcohol-induced renal damage mostly through the inhibition of pro-apoptotic mediators including caspase enzymes and Bax/Bcl-2 signaling, as well as pro-inflammatory cytokines production (Rezaee-Khorasany et al., 2019). Crocin has demonstrated nephroprotective properties in an animal model of diabetes-induced nephrotoxicity (**Table 2**). Aside from general antioxidant properties (Erdemli et al., 2017), this compound could decrease hyperurocemia *via* the inhibition of xanthine oxidase (Altinoz et al., 2015). Moreover, a clinical trial using 100 mg/day of saffron in healthy subjects showed this plant to have a short-term immunopotentiation, considering the altered levels of immunoglobulins and the leukocytes count (Kianbakht and Ghazavi, 2011).

## Discussion and Conclusions

This paper has reviewed the pharmacological mechanisms of medicinal plants with possible beneficial effects on SARS-CoV-2 infection and related organ damage based on the suggestions of TPM. Most of the medicinal plants included in this review show multitargeted activity and protective mechanisms in the tissues damaged in SARS-CoV-2 infection.

**Figure 1:**
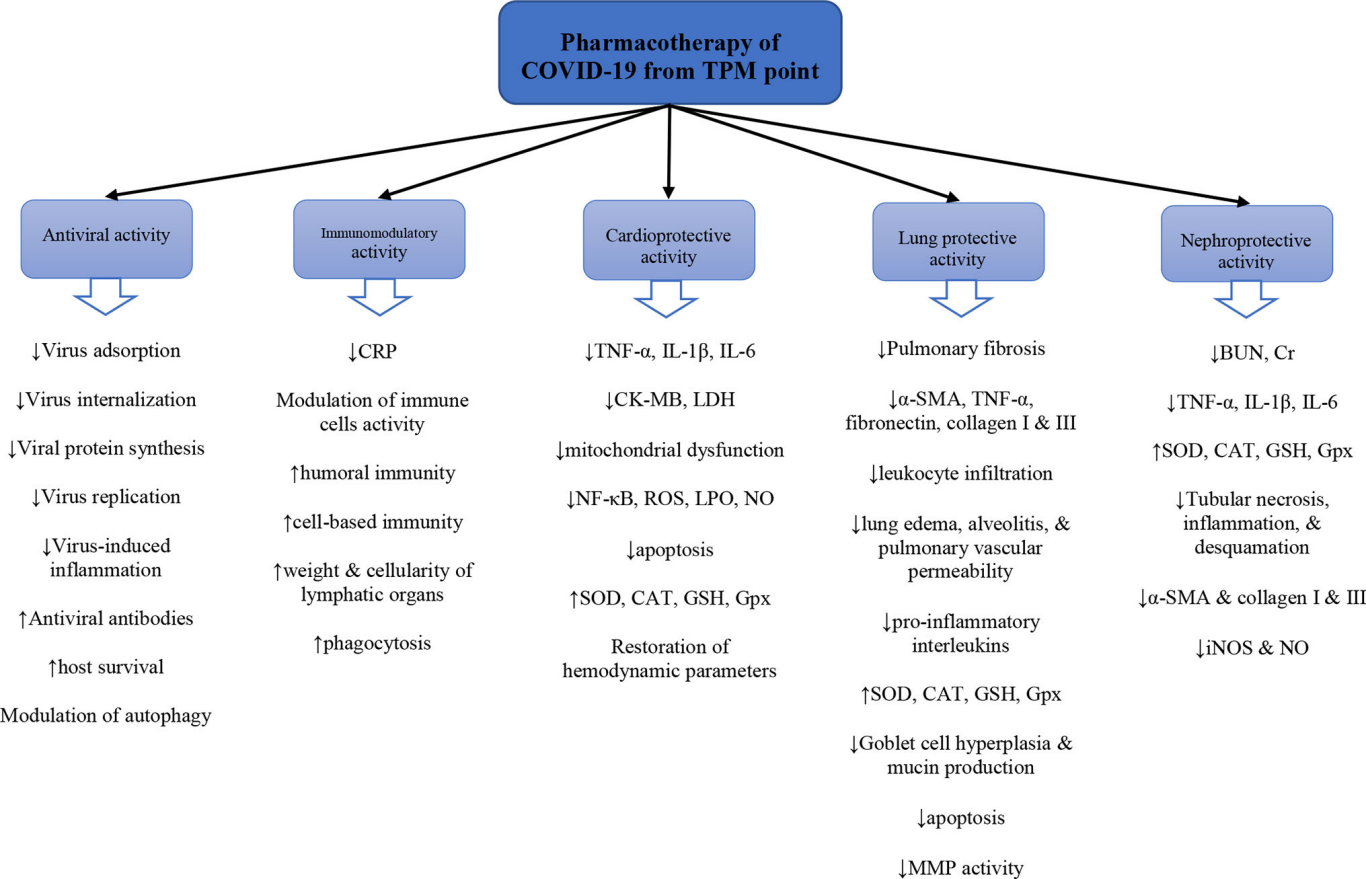
Mechanisms of medicinal plants introduced based on traditional Persian medicine for the management of SARS-CoV-2 infection.

The most important effects of the medicinal plants and their isolated phytochemicals include anti-inflammatory activity and antioxidant properties (**Figure 1**). These two mechanisms comprise several cellular and subcellular pathways such as NF-κB, Nrf2, pro-inflammatory and anti-inflammatory cytokines balance, and endogenous enzymatic/non-enzymatic antioxidant defense mechanisms. It should be considered that such mechanisms are general cytoprotective cascades observed in nearly all body organs and that the beneficial effects of phytochemicals in one tissue can be extrapolated to other tissues. For instance, if a compound has shown a stimulatory effect on Nrf2 signaling in myocardial cells, the same activity could be expected in lung tissue or kidney tubules. This is in line with the holistic view of TPM, which encompasses the idea that treatments should improve the overall health of the human body as a whole, instead of focusing on the damaged organ. It is believed in TPM that reinforcement of the body’s inner power is one of the best ways to combat a disease. Most of the discussed medicinal plants stimulate cytoprotective mechanisms to face pathogenic factors, which can be considered as equivalent to these ideas of improving the body’s inner power.

One of the key pro-inflammatory cytokines affecting clinical manifestations of COVID-19 is IL-6. In patients with COVID-19, organ failures have been observed and the subsequent deaths of many patients are often due to cytokine storm, an exaggerated inflammatory response (Mehta et al., 2020). IL-6 has a major role in this event and the IL-6 inhibitor tociluzumab (Actemra^®^) has been evaluated in several clinical studies in patients. Another systematic review has also discussed the value of this agent in treating COVID-19, highlighting that current evidence suggests that the drug could be beneficial in this infection (Alzghari and Acuña, 2020). Another important cytokine in this infection is TNF-α and the inhibitors of this cytokine are also currently being assessed as therapeutic options for SARS-CoV-2 patients (Feldmann et al., 2020). A considerable number of the plants discussed in this present review have been shown to reduce IL-6 and TNF-α levels and/or activities (**Table 2**). Major phytochemicals of these plants should be further investigated in the design and development of plant-derived cytokine inhibitors in future studies.

Edible plants, particularly grape, jujube fruit, amla, and damask rose, are well-known and used medicinally every day by people all over the world, which indicates that they are safe. Such plants can be prepared as a juice or in a standardized liquid dosage form for COVID-19 patients. Where the guidelines on effective drugs for treating this infection vary day-by-day, increasing amounts of new data are being released from ongoing clinical trials regarding the pros and cons of the medicines recommended. In such a situation, physicians may be willing to stay on the safe side and avoid using multi-ingredient complementary medicinal preparations with several possible side effects/drug interactions. As a more acceptable and safer approach, natural tonics that are based on these popular food plants could be recommended as part of the healthy daily diet for people prone to or who are vulnerable to the disease. This includes healthcare providers caring for COVID-19 patients in hospitals, the family members of the patients in home-quarantine, and people with underlying diseases such as cardiovascular problems or diabetes who are more vulnerable to this infection. Studies on healthy populations are encouraged due to the lower risk of adverse effects and the fact that they would indicate the safety and efficacy of natural products, with use in patients as the second step in developing these adjunct therapies.

On the other hand, some phytochemicals have revealed direct antiviral activity *via* blockade of different stages of the virus life cycle including fusion, replication, protein synthesis, and viral particle release from the host cells. Although the life cycle and target proteins of SARS-CoV-2 may differ from the assessed viruses to some extent, there would be similarities that are worth assessing in terms of the antiviral activity of these compounds against the virus. As has been reported in virtual screenings (Zhang et al., 2020a), some of these compounds have shown significant interactions with SARS-CoV-2 structures, further confirming their antiviral activity. In this regard, molecular docking analyses would help in selecting structures with the highest binding affinity and the ability to inhibit viral enzymes. The virtually selected antiviral phytochemicals can then be evaluated in cell-based and animal studies. Likewise, they can be considered as molecular backbones in the design of new semisynthetic structures and in creating new compounds with higher safety and antiviral efficacy.

Some of the included compounds such as quercetin, pyrogallol, and fructan polysaccharides are considered ubiquitous, i.e. they can be found in several foods and spices of the human diet and do not belong to a specific medicinal plant. Despite the abundance of these compounds, their pharmacological activity cannot be denied and further interpretation of the results of studies is of great importance. For example, in an *in vitro* study by Mahmood et al. (1996) quercetin showed the highest antiviral activity against HIV amongst several evaluated phytochemicals. This does not mean that any quercetin-containing plant can be effective against viral infection. Instead, the effective dose of this compound should be measured in preclinical studies and a dose translation calculation is needed to suggest accurate human doses of quercetin and to observe related pharmacological activity.

An important limitation of the studies included in this review is that many of them lacked a positive control group. Some of the reports used dexamethasone as a standard anti-inflammatory agent in antiviral evaluations, however, most of the studies did not consider a standard treatment. This methodological problem makes any judgment regarding the potency of the assessed materials difficult. Some antiviral phytochemicals have significantly lower SI compared with conventional antiviral agents which excludes them from further assessment. Lack of a positive control makes such comparisons difficult since SI reported in other studies do not provide an accurate comparison due to these methodological differences. The same problem exists in the evaluation of the other pharmacological properties of the plants. Thus, future studies should consider the use of a gold standard drug for a better presentation of the potency of test agents.

One of the concerns in the clinical use of medicinal plants is the possibility of herb-drug interaction. Phytochemicals can have inducing (Soleymani et al., 2017) or inhibitory effects (Bahramsoltani et al., 2017) on drug metabolizing enzymes such as hepatic cytochrome P450 and intestinal P-glycoprotein and thus, can affect the pharmacokinetics of conventional drugs. Consequently, the serum level of conventional antiviral agents administered to COVID-19 patients may be affected by concomitant administration of herbal supplements. Such pharmacokinetic interactions may increase serum level and adverse effects, or decrease serum concentration and clinical response, both of which can have life-threatening outcomes in patients.

Another discussion can be raised concerning the plants that contain ACE inhibitor compounds. ACE inhibitors do not have a direct effect on SARS-CoV-2 but they can increase the expression of the ACE2 receptors. This receptor acts as a co-receptor in the invasion of the virus and thus, a hypothesis is formed that ACE inhibitors may increase susceptibility to SARS-CoV-2 (Danser et al., 2020).

Most of the studies included in this review were animal and cell-based evaluations, providing only foundational evidence for the efficacy of the aforementioned plants in human studies. Only six clinical studies were considered in this paper and higher levels of evidence are essential to examine the medicinal suggestions of TPM in clinical settings. Although there are several ongoing clinical trials on the effect of TPM formulas on COVID-19, no published data is available. Despite the challenges in decision-making for the clinical assessment of natural products in SARS-CoV-2, high-quality trials have evaluated the effect of such products with valuable outcomes (Yang et al., 2020), with over fifty clinical trials designed to assess the effect of self-made and commercial TCM formulas. The National Health Commission of China has also added some TCM recommendations to the latest guidelines for the management of COVID-19 patients, even in severe cases (Yang et al., 2020). Thus, the trial design and inclusion/exclusion criteria for patients, as well as drug preparation can be defined in a way that is comprehensively approved. Natural compounds may have some advantages in comparison to conventional drugs. For instance, there are concerns about the currently available TNF-α inhibitors because they can suppress the immune system, leading to secondary bacterial/viral infections (Feldmann et al., 2020). On the other hand, a remarkable number of natural antiviral compounds have immunomodulatory and antibacterial properties. Further studies in healthy individuals that assess the prophylactic activity of these supplements as well as trials in patients with less severe symptoms would pave the way for further clinical evaluations.

In conclusion, medicinal plants have great potential value and can be recommended for treatment of COVID-19 based on the therapeutic approaches of TPM, several of which have also been confirmed by pharmacological studies in modern medicine. The currently available data, regarding these medicinal plants, provide foundational evidence. Future preclinical mechanistic studies as well as clinical trials are necessary to confirm the safety and efficacy of these plants for the management of SARS-CoV-2 infection.

## Author Contributions

All authors contributed to the article and approved the submitted version.

## Funding

This work is partially supported by the Tehran University of Medical Sciences (Grant No. 99-1-147-47262).

## Conflict of Interest

The authors declare that the research was conducted in the absence of any commercial or financial relationships that could be construed as a potential conflict of interest.
